# Molecular dissection studies of *TAC1*, a transcription activator of *Candida* drug resistance genes of the human pathogenic fungus *Candida albicans*

**DOI:** 10.3389/fmicb.2023.994873

**Published:** 2023-07-12

**Authors:** Tushar Jain, Pankaj Mishra, Sushil Kumar, Gautam Panda, Dibyendu Banerjee

**Affiliations:** ^1^Academy of Scientific and Innovative Research (AcSIR), Ghaziabad, Lucknow, Uttar Pradesh, India; ^2^CSIR-Central Drug Research Institute, Lucknow, Uttar Pradesh, India

**Keywords:** *TAC1*, *CDR1*, *CDR2*, *Candida albicans*, MDR, azole drug resistance, xenobiotic response, drug response element

## Abstract

The up-regulation of ABC transporters Cdr1p and Cdr2p that efflux antifungal azole drugs are a leading cause of Multi-Drug Resistance (MDR) in the white fungus *Candida albicans*. *C. albicans* was reported to infect patients following the recent Covid-19 pandemic after they were given steroids for recovery. Previously, the *TAC1* gene was identified as the transcriptional activator of *Candida* drug resistance genes (*CDR1* and *CDR2*) and has no known human homologs. This makes it a good target for the development of novel antifungals. We, therefore, carried out the molecular dissection study of *TAC1* to understand the functional regulation of the ABC transporter genes (*CDR1* and *CDR2*) under its control. The N-terminal DNA Binding Domain (DBD) of Tac1p interacts with the Drug Responsive Element (DRE) present in the upstream promoter region of *CDR1* and *CDR2* genes of *C. albicans*. The interaction between DBD and DRE recruits Tac1p to the promoter of *CDR* genes. The C-terminal Acidic Activation Domain (AAD) of Tac1p interacts with the TATA box Binding Protein (TBP) and thus recruits TBP to the TATA box of *CDR1* and *CDR2* genes. Taking a cue from a previous study involving a *TAC1* deletion strain that suggested that Tac1p acts as a xenobiotic receptor, in this study, we identified that the Middle Homology Region (MHR) of Tac1p acts as a probable xenobiotic binding domain (XBD) which plays an important role in *Candida* drug resistance. In addition, we studied the role of Tac1p in the regulation of some lipid profiling genes and stress response genes since they also contain the DRE consensus sequence and found that some of them can respond to xenobiotic stimuli.

## Introduction

*Candida albicans* is a major cause of opportunistic and nosocomial infections in humans. Although a commensal in the human body, *Candida* can quickly turn pathogenic if the host immune system is compromised due to other infections such as HIV, steroid treatment, or due to chemotherapy or radiation therapy. The antifungals present today can become quickly ineffective due to the MDR (Multidrug Resistance) phenomenon whereby all drugs are pumped out of the fungal cells by ABC transporter pumps present in their cell membrane. The discovery of new antifungals has not kept pace with the clinical development of drug resistance. This is because, being an eukaryote, the number of unique drug targets available are quite limited (Roemer and Krysan, 2014). The commonly exploited targets include the enzymes of the ergosterol biosynthetic pathway and β-1,3 glucan synthase which are components of the fungal cell membrane and cell wall, respectively (Akins and Sobel, 2017). Only 3 classes of antifungals are currently in use *viz.* Azoles, Polyenes, and the more recently discovered Echinocandins (Robbins et al., 2016; Ksiezopolska and Gabaldón, 2018). Unfortunately, with the subsequent development of Multi-Drug Resistance (MDR) in species like *C. albicans* and *C. glabrata*, no effective treatment measures are available (Tanwar et al., 2014). Multidrug resistance is the simultaneous development of drug tolerance due to a few or even just one genetic mutation (Paul and Moye-Rowley, 2014). The ABC transporter-encoding *CDR1* gene and *CDR2* are the two genes most thoroughly studied for their roles in multidrug resistance in *C. albicans* (Prasad et al., 2015) and MDR1 locus encoding a major facilitator superfamily (MFS) protein (Fling et al., 1991). Because of the limited availability of unique targets, we plan to inhibit the regulatory circuit responsible for the upregulation of ABC transporters; this can potentiate the activity of currently available antifungal drugs by reducing their efflux. In order to target the regulatory circuit of MDR, its molecular regulation must be fully understood. The mode of development of antifungal drug resistance was studied in detail in the recent past and different research groups have identified the role of efflux pumps including ATP-Binding Cassette (ABC) and Major Facilitator Superfamily (MFS) transporters in the development of MDR (Costa et al., 2014; Ren et al., 2014; Cowen et al., 2015; Prasad et al., 2015).

One fungal-specific transcription factor Tac1p (Transcription activator of *CDR* genes) was discovered and found to be responsible for the up-regulation of ABC transporters *Candida* drug resistance 1 protein (Cdr1p) and *Candida* drug resistance 2 protein (Cdr2p), present on the cell membrane of *C. albicans* (Coste et al., 2004). These ABC transporters are responsible for the efflux of different classes of structurally unrelated chemical moieties leading to MDR in *Candida* sp. (Lage, 2003; Holmes et al., 2008). Cdr1p and Cdr2p transporters are encoded by *CDR1* and *CDR2* genes which are located on chromosome 3 of *C. albicans* (Candida Genome Database, n.d.). Transient up-regulation of *CDR1* and *CDR2* genes in *C. albicans* upon xenobiotic induction has identified various responsive elements present in the upstream promoter regions of *CDR* genes (Kusch et al., 2004; Kofla et al., 2011). One such xenobiotic, Fluphenazine was identified to transiently up-regulate *CDR* gene expression due to the presence of DRE in the upstream promoter region (−380 base pair) of these genes (De Micheli et al., 2002). De Micheli et al., in the year 2002, observed that these DRE’s might be putative zinc cluster binding sites since they contained CGG triplets in a direct repeat orientation (De Micheli et al., 2002). A genome-wide search for putative proteins containing the highly conserved Zn2-Cys6 motif mapped the *TAC1* gene to a region near the mating-type locus that was previously linked to azole resistance in a few clinical isolates (Coste et al., 2006). However, the detailed molecular regulation of *CDR1* and *CDR2* genes by Tac1p is not yet fully understood.

In this context, we conducted a domain dissection study of Tac1p and studied its interaction with various partner proteins such as (1) the promoter region of the *CDR* gene, (2) the putative cofactor (TATA-box Binding Protein), and (3) a xenobiotic (Fluphenazine). *TAC1* (2,946 nucleotides) houses three domains, an N-terminal DNA Binding Domain (DBD 1–1,320 nucleotides); a C-terminal Acidic Activation Domain (AAD 1354-2946 nucleotides), and a Middle Homology Region (MHR 1321-1653 nucleotides). The role of individual domains to bring about transcription of *CDR* genes and their transient upregulation was studied in detail using Electrophoretic Mobility Shift Assay (EMSA), GST-Pull down assay, Yeast-2-hybrid assay, semi qRT-PCR, and domain deletion studies. With the concerted use of computational and biological experiments, we established the structural assembly of *TAC1-DBD* and DRE. In addition to that, we also checked the expression of lipid profiling genes as well as stress response genes to check whether *TAC1* plays any role in the expression of these genes besides the regulation of *CDR1* and *CDR2* genes. As per the previous report (De Micheli et al., 2002), *CDR1* and *CDR2* contain a cis-acting element, which is well known as DRE (Drug response element) that is important for their upregulation after drug exposure, especially in azole-resistant isolates of *C. albicans*. Therefore, we have selected only those genes which have a DNA sequence similar to the 21 base-pair DRE consensus sequence. For that, we have performed sequence-specific multiple sequence alignment of each selected gene with DRE consensus sequence by using the online Clustal Omega Multiple Sequence Alignment tool. Finally, we selected only those genes which showed ≥55% sequence similarity with the DRE sequence of *CDR1* and *CDR2* genes (Hameed and Fatima, 2013).

## Results

### Homology modeling of Tac1-DBD

**Figure 1:**
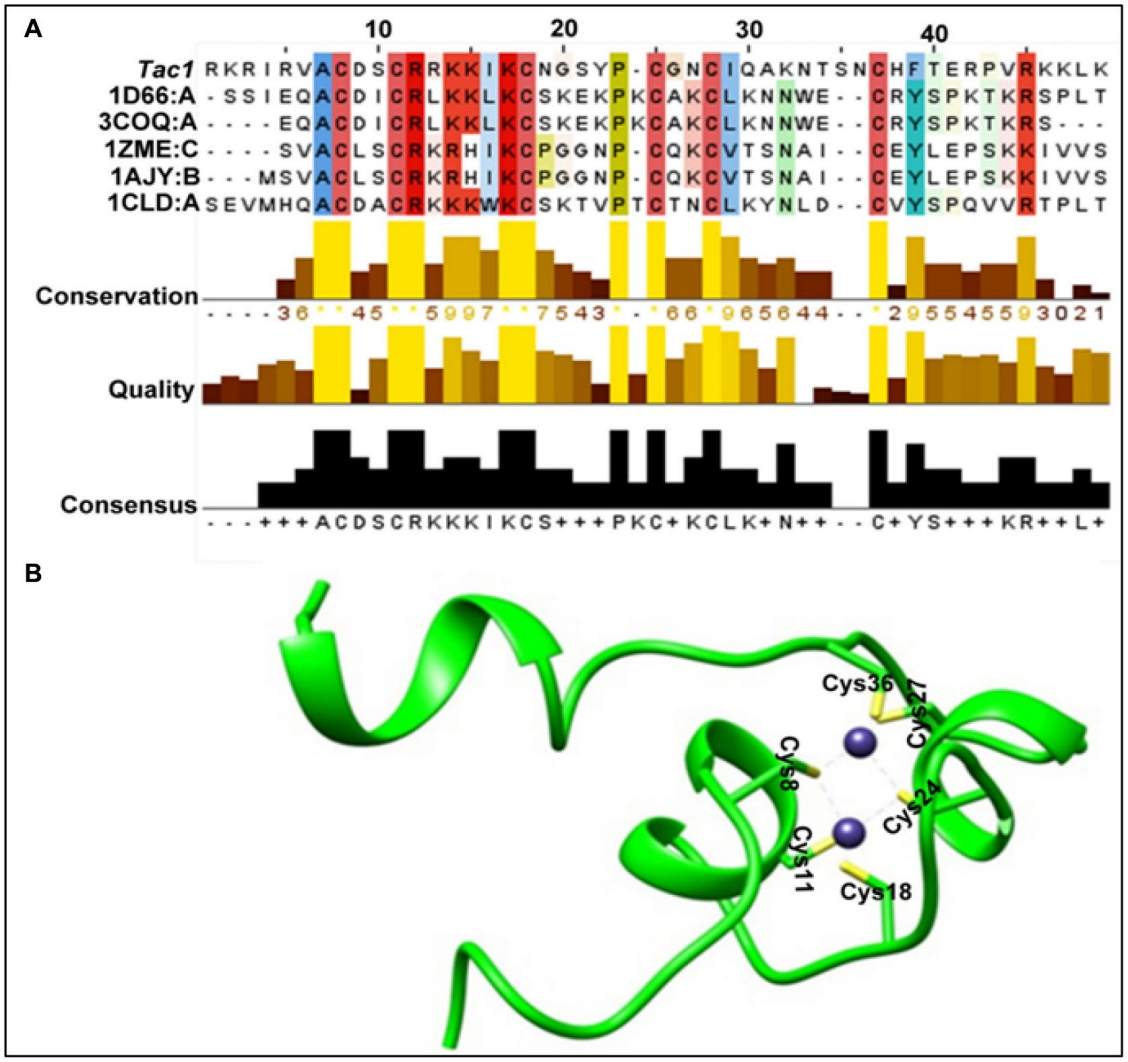
DBD homology model. **(A)** Overall characteristics of the selected templates and DBD sequence. **(B)** Structure of the representative DBD homology model (green ribbon representation). Zinc atoms are shown as blue spheres. Cysteine residues (In stick representation) are positioned in coordination with two zinc atoms.

In the absence of any prior structural information, we carried out homology modeling of this protein. The FASTA sequence search of the protein data bank led us to identify the closest homolog of Tac1-DBD as GAL4 of *Saccharomyces cerevisiae* (PDB ID 1D66, query coverage, 91%, e-value, 0.018 and sequence identity, 40%). However, we noticed three missing residues in the crystal structure. On further analysis of the sequence clusters from BLAST search, four other homologs were identified (PDB id 3COQ, 1ZME, 1AJY, and 1CLD) and used to perform the multiple templates based homology modeling in Modeler 9.13 (Webb and Sali, 2016). Figure 1A represents the overall characteristics of the selected templates. In principle, broad query coverage, low e-value, and high sequence identity are the matrices for the reliability of the homology model, and in our case, these criteria were satisfied. Initially, we selected the top 10 models of DBD based on the Modeler default score *viz.* DOPE, MOLPDF, and GA341 score (Chen et al., 2018). On further optimization of the individual structures via energy minimization with CHARMM27 all-atom force field (Vanommeslaeghe et al., 2010) to remove any steric clashes and subsequent protein structural validation through Ramachandran plot (Hooft et al., 1997), ProSA plot (Wiederstein and Sippl, 2007), ERRAT plot (Messaoudi et al., 2013) has resulted in the selection of the best homology model. Figure 1B represents the validity results of the selected DBD homology model. The secondary structure of DBD determined by STRIDE (Heinig and Frishman, 2004) also confirmed the optimized model as a suitable structure for further evaluations (Figure 1B). It is worth mentioning here that the superimposition of the obtained homology model and corresponding GAL4 protein displayed the lowest RMSD value of 0.689 Å, suggesting the stability and robustness of the model. The DBD of *C. albicans* belongs to the Zn_2_Cys_6_ family in which two zinc ions coordinate with six cysteine residues and play a crucial role in the DRE recognition process in addition to their primary role of stabilizing the α-helix of DBD (MacPherson et al., 2006). Six cysteine residues were coupled to two Zn atoms in our system, and the binding was optimized by using UCSF chimera (UCSF Chimera Home Page, n.d.). The final modeled structure with optimized Zn^2+^ geometry was marked as DBD (Figure 1B) and utilized in further studies.

### Structural assembly of Tac1-DBD and DRE of *CDR* genes

**Figure 2:**
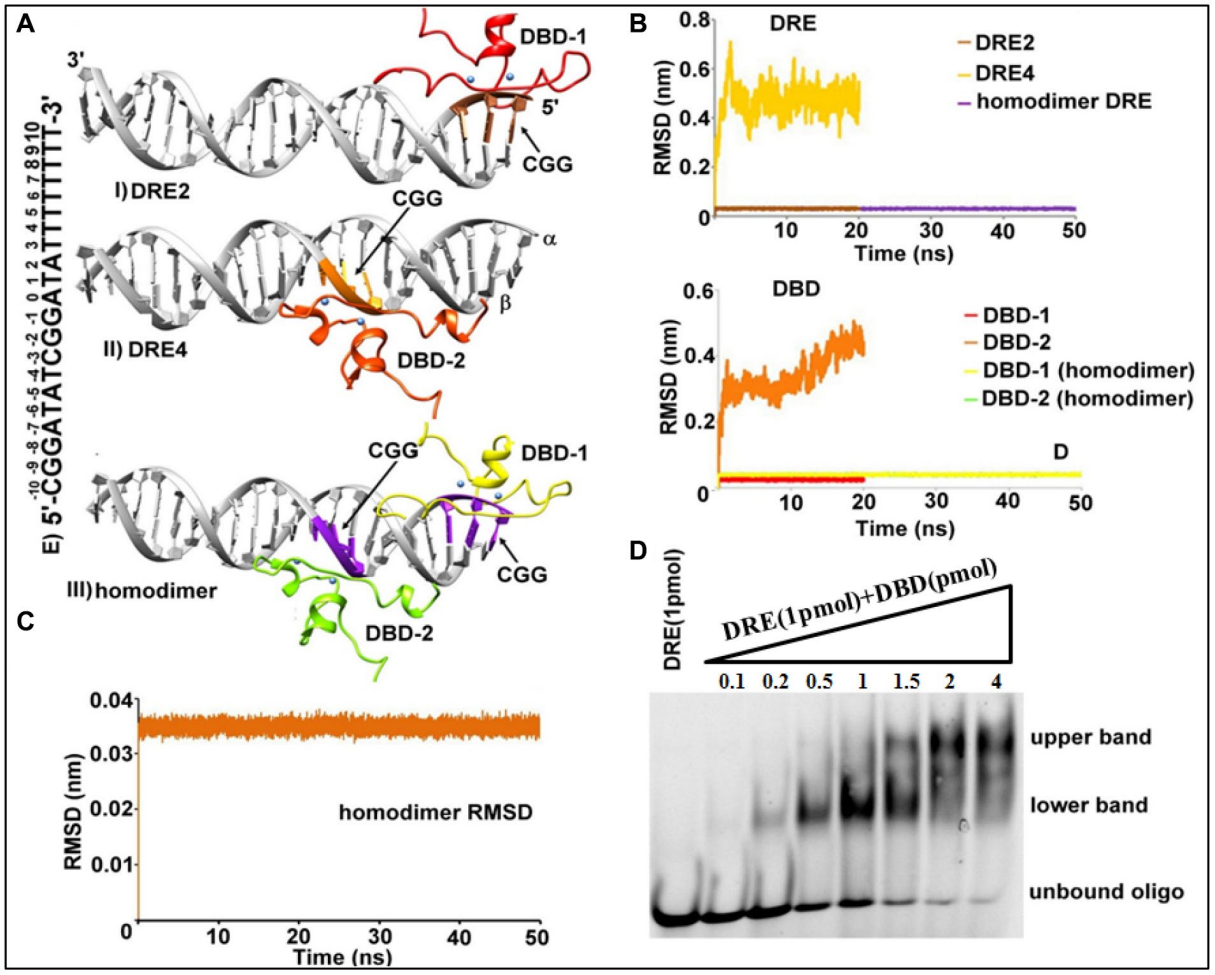
Structural assembly of *TAC1* DBD-DRE in *C. albicans*. **(A)** DBD-DRE docking complexes, (I) DRE2 system, (II) DRE4 system, and (III) homodimer complex. Please refer to the text for the significance of these complexes. DRE (Grey color) and DBD are shown in the ribbon representation. **(B)** Backbone atoms RMSD of DRE (Above) and DBD (Below) in DRE2, DRE4, and homodimer complex. Protein color corresponds to the color of the RMSD plot for the respective regions. **(C)** Backbone atoms RMSD of the homodimer complex. **(D)** Electrophoretic mobility shift assay. 1 pmol of 5’ FAM-labeled DRE was incubated with increasing concentrations (0.1–4 pmol) of *TAC1-DBD*.

Distance-dependent HADDOCK program was used for protein-DNA docking (De Vries et al., 2010). Under this approach, the canonical DNA structure was obtained utilizing the 3D-DART server (van Dijk and Bonvin, 2009) with the DRE sequence (5′-CGGATATCGGATATTTTTTTT-3′) as input. In earlier work, preferential binding of DBD to the CGG cluster of DRE was suggested (Liu et al., 2007). A gene cluster is a collection of two or more genes that are commonly found close to one another in an organism’s DNA and that together encode comparable polypeptides, or proteins, with a common function (Znaidi et al., 2007) *TAC1* gene of *C. albicans* is found on zinc cluster region of chromosome 5 (Coste et al., 2004). Notably, in *C. albicans,* these clusters essentially lie in between the first 10 base pairs (Figure 2A), and to decipher the most plausible mode of their interaction, we utilized four different combinations of residues *viz.* ± 10 to ±7 (CGG), ± 3 to ±1 (CGG), ± 10 to ±7 (CGG), ± 3 to ±1 (CGG), and ± 10 to ±1 (CGGATATCGG) designated as DRE1-4 respectively, as the restraints for docking. Nucleotides lying within 6.5 Å of the active nucleotides were automatically considered as passive residues. The entire sequence of DBD was assigned as the active site. A total of 18 clusters (Table 1) were retrieved as output, of which we selected the best putative complex structure based on negative *z-*score value, highest cluster size, and visual inspections. Two representative structures were selected from DRE2 (Figure 2A-I; Table 1) and DRE4 (Figure 2A-II; Table 1) clusters. Further, we observed that Zn_2_Cys_6_ primarily binds to the CGG region, and no preferential interactions were observed within the ±7 to ±4 (ATAT) region of DRE, hence endorsing our homology model.

**Table 1:** HADDOCK-based docking output of DBD-DRE samples.

± 10 to ±7 (CGG) DRE1		± 10 to ±7 (CGG) - ± 3 to ±1 (CGG) DRE2		± 10 to ±1 (CGGATATCGG) DRE3		± 3 to ±1 (CGG) DRE4	
Cluster size	*z*-score	Cluster size	*z*-score	Cluster size	*z*-score	Cluster size	*z*-score
105	0.3	89	−1.6	48	0.4	104	−1.3
20	1.0	33	0.8	33	−0.9	21	−0.7
14	−1.4	22	1.1	25	−1.2	11	−0.2
		17	−0.7	24	1.6	10	1.6
		8	0.4	13	0.1	9	0.7

### Tac1-DBD exists as a homodimer

It is well-known that the majority of zinc finger proteins interact with DNA to perform their function in transcriptional and translational processes and these interactions may lead to the existence of a monomeric, homo-dimeric, or hetero-dimeric arrangement. As evident from Table 1, the docking output from DRE4 resulted in the highest cluster size and negative *z*-score, which indicated that Tac1 exists as a homodimer. Macpherson et al. summarized the different kinds of DBD Zn_2_Cys_6_ regulators based on the available crystal structures (MacPherson et al., 2006). On visual inspection of docking-based selected systems of DRE2 and DRE4, it was apparent that a similar kind of system that exists in Hap1p (PDB id 1HWT), which binds to direct repeats (Hellauer et al., 1996), might exist for Tac1p. These observations compelled us to crosscheck the possibilities of the existence of a homodimer-like arrangement, for which we relied on classical MD simulations of the individual systems. DBD-DRE complexes from DRE2 and DRE4 were initially subjected to 20 ns MD simulations with our expectations that the putative complex will be those having stable orientations during simulations. It was evident from the simulation results that DRE2-related complexes were highly stable during simulations while DRE4 was relatively unstable as incidental from their overall RMSD (Root Mean Square Deviation) values (~ 0.08 Å for DRE2 and 0.5 Å for DRE4) (Figure 2B). The difference between a protein’s backbones from its initial structural conformation to its final position is measured using the RMSD. However, when the complexes were superimposed to form a homodimer and further subjected to MD simulations for 50 ns, it showed higher stability compared to the monomers (Figures 2B,C). Over the course of simulations, backbone RMSD for DRE2 and DRE4 was ~0.08 Å while RMSD of the P atom of DRE was also ~0.08 Å (Figure 2C). The RMSD of the overall system was ~0.035 Å and as such, the value of this range suggests that the system was relatively immobile and hence most stable.

To validate these observations, we further assessed the binding of Tac1 DBD with the DRE in the lab using a gel-based electrophoretic mobility shift assay (EMSA) (Figure 2D). 1 pmol of 5’ FAM-labeled DRE was incubated with increasing concentrations (0.1–4 pmol) of purified Tac1 DBD protein and run on a native gel. Interestingly two different band patterns were observed and it was inferred that at lower protein concentrations Tac1 DBD binds to DRE as a monomer in which case, a lower band on the gel was observed. With the subsequent increase in protein concentration, an upper band corresponding to a higher oligomeric form of the bound Tac1 DBD - DRE complex was observed (Figure 2D). The stoichiometric analysis of DNA: protein concentration of the oligomeric state was found to be 1:2, which corresponds to a dimer as suggested by *in silico* experiments. Hence, based on the results from both computational and gel-based experiments, a homodimer arrangement with two monomers of protein bound to one oligo is proposed as the most plausible architecture for the DBD-DRE complex of *C. albicans.*

### Tac1-AAD interacts with TATA-binding protein (TBP)

**Figure 3:**
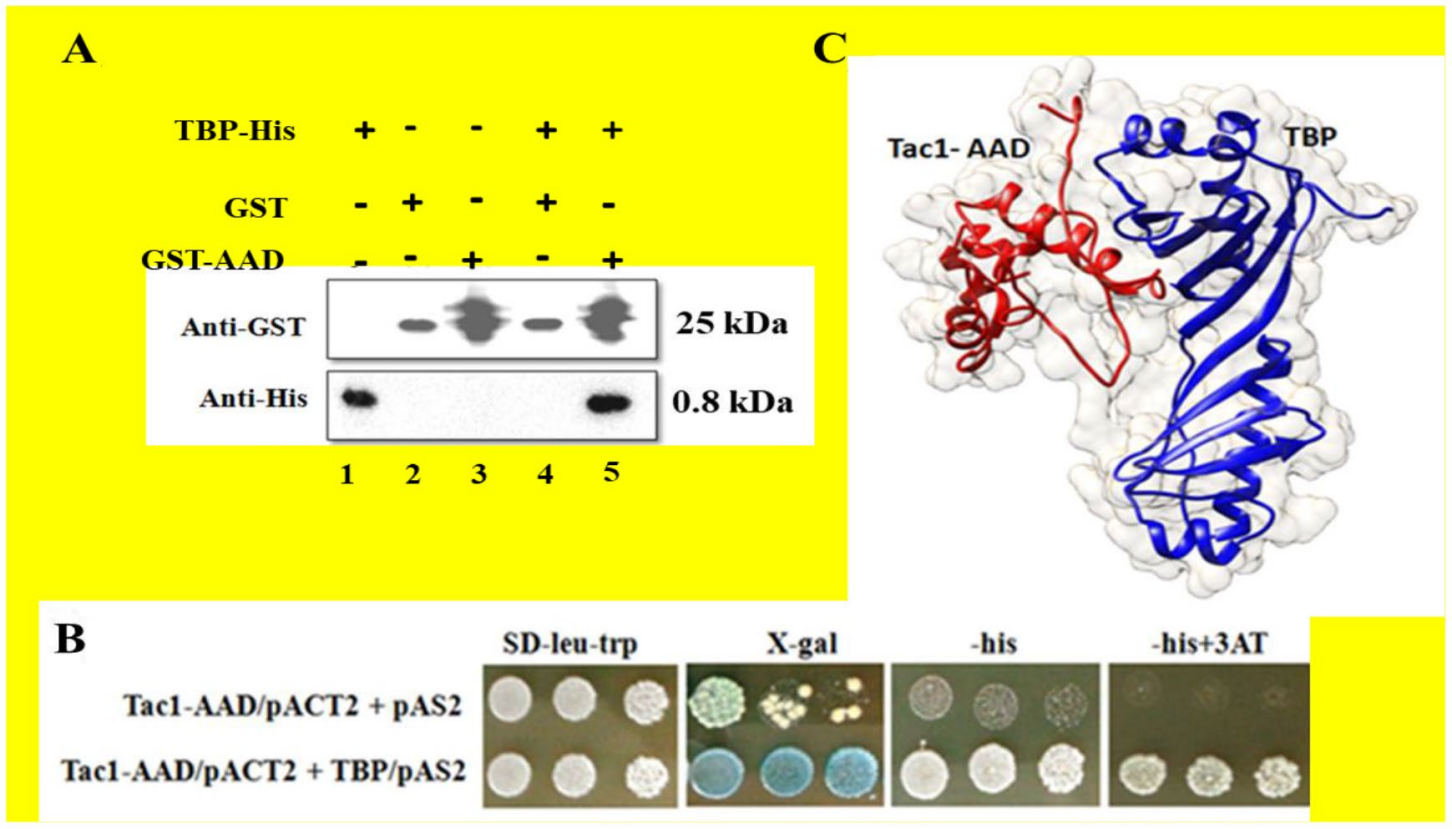
The interaction between the AAD domain of Tac1p and TBP was studied using GST pull-down and Yeast 2 hybrid assays. **(A)** Represents immuno-blots of purified protein and eluates after GST pull-down, detected using Anti-GST and Anti-His antibodies, respectively. **(B)** Colonies obtained from Yeast 2 hybrid assay were plated on minimal media lacking leucine and tryptophan. LacZ and *His* reporter genes were used to establish interaction for which SD plate containing X-gal and lacking histidine were employed. 3AT was added to prevent leaky expression of the *HIS* gene. **(C)** Model depicting the interaction of *TAC1-AAD* and TBP.

The activation domain to Tac1p (AAD) was expressed in *Escherichia coli* strain BL21-DE3 as part of a fusion protein (Molecular weight 33 kDa) with GST-tag and purified using Glutathione Sepharose 4B beads for GST-affinity chromatography. A putative cofactor, *C. albicans* TBP was expressed in the same host as a His-tagged fusion protein (Molecular weight 30 KDa) and purified using Ni-NTA affinity chromatography. To establish whether AAD directly binds to TBP, a GST pull-down assay was performed, and the interaction was further confirmed using a Yeast-2-hybrid assay. Pull-down assay established a strong direct interaction between AAD and TBP. To eliminate the possibility of electrostatic interaction, both proteins were incubated with Glutathione Sepharose 4B beads followed by washing with a high salt concentration (500 mM NaCl) buffer. Bound proteins were eluted using 10 mM-reduced glutathione and run on 12% SDS PAGE. AAD and TBP were detected using immune-blotting with Anti-GST and Anti-His antibodies, respectively. Figure 3A (lane 5) shows the co-elution of AAD and TBP whereas TBP was not detected in Glutathione Sepharose 4B beads control (lane 4), indicating a strong interaction between the two proteins. Activation domain-containing acidic amino acid residues were reported to be flexible and amenable to degradation upon *in-vitro* purification. This resulted in several closely spaced bands upon immune-blotting with an Anti-GST antibody. Hence, we resorted to an *in-vivo* Yeast-2-hybrid system to further confirm the interaction. The AAD-pACT2 construct was cloned replacing Gal4-AD with Tac1-AAD. The TBP-pAS2 construct was cloned with N-terminal Gal4-DBD. Both the constructs were transformed in *S. cerevisiae* strain PJ69-4A. The transformants were able to grow on minimal media (SD-trp-leu) lacking histidine. 3AT prevented the leaky expression of the *HIS* marker gene. *LacZ* reporter assay resulted in the formation of blue colonies in the presence of X-Gal establishing *in-vivo* interaction between AAD and TBP (Figure 3B). Model depicting the interaction of Tac1-AAD and TBP was shown in Figure 3C.

### Tac1-MHR is a xenobiotic binding domain

**Figure 4:**
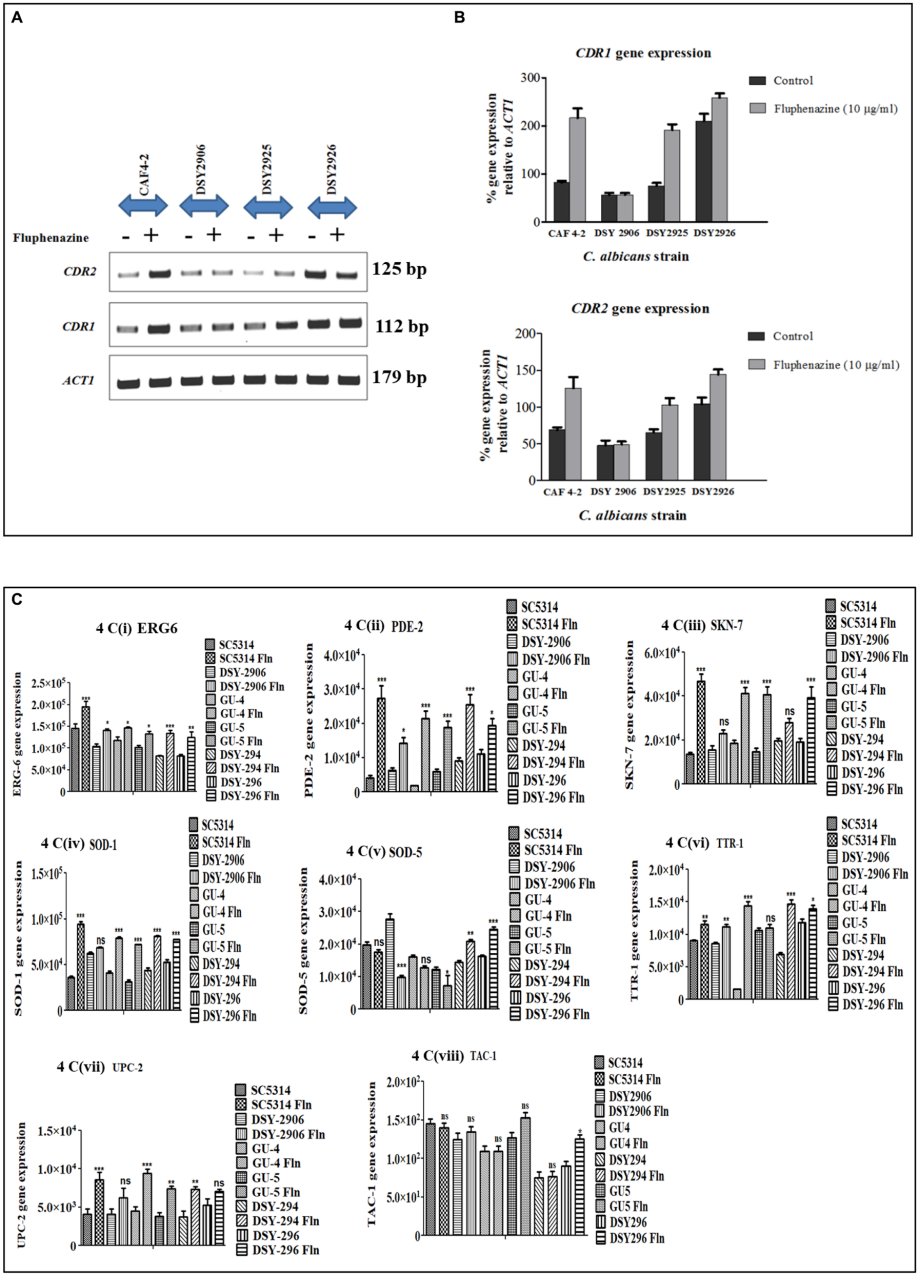
Semi-quantitative PCR of ABC transporter genes, lipid profiling genes, and stress response genes of *C. albicans* was conducted. *ACT1* was used as an internal control. **(A)** Agarose gel images of *ACT1, CDR1,* and *CDR2* gene expression in different strains of *C. albicans viz.* CAF4-2, DSY2906, DSY2925, and DSY2926 in the absence and presence of Fluphenazine. **(B)** Bar graph showing percentage change in expression of *CDR1* and *CDR2* genes after densitometric analysis. Values shown are mean ± SEM for three independent experiments. *p < 0.05; **p < 0.01 when compared with vehicle control using Student’s *t*-test. **(C)** Bar graph showing the expression of lipid profiling gene (*ERG6*) and stress response genes (*PDE-2*, *SKN-7*, *SOD-1*, *SOD-5*, *TTR-1*, *UPC-2*, *TAC-1*). Values shown are mean ± SEM for three independent experiments after densitometric analysis. *p < 0.05; **p < 0.01, ***p < 0.0001 when we compared uninduced strains with Fluphenazine-induced strains using One-way analysis of variance (ANOVA).

Transient up-regulation of *CDR* gene expression upon induction with the xenobiotic Fluphenazine was reported previously (Karababa et al., 2004). A 21 bp consensus DRE sequence present −380 bp upstream in the promoter region of *CDR* genes was responsible for xenobiotic-induced up-regulation (Prasad et al., 2012). In our experiments, we have already shown that Tac1-DBD binds to the DRE of *CDR* genes. Further, to investigate the role of Tac1p in xenobiotic-induced up-regulation of target genes, strains *CAF 4–2* (containing wild type *TAC1*); *DSY2906* (*TAC1* deletion); *DSY2925* (wild type *TAC1* revertant), and *DSY2926* (GOF *TAC1* revertant) were employed (Table 2). All the strains were grown overnight in 10 mL YPD (Yeast Extract–Peptone–Dextrose) broth media, then diluted to a cell density of 1 × 10^7^ cells/mL and grown for an additional 2 h. 10 μg/mL Fluphenazine was then added to the cultures and incubated for 20 min. After 20 min, cells were harvested by centrifugation and total RNA was isolated using RNeasy mini kit (Qiagen, India). *CDR1* and *CDR2* mRNA levels were determined using Semi-Quantitative PCR. As evident from Figure 4, strain *DSY2906* showed no change in mRNA expression levels of *CDR1* and *CDR2* genes upon Fluphenazine induction. This preliminary study suggested that Tac1p might act as a xenobiotic receptor.

**Table 2:** Different strains of *C. albicans viz*. CAF4-2, DSY2906, DSY2925, and DSY2926 and their genotype that was used in Figures 4A, B (i) for checking the expression of *ACT1, CDR1,* and *CDR2* gene expression in the absence and presence of Fluphenazine.

S.No.	Strains of *C. albicans*	Genotype
1.	CAF4-2	*URA*3Δ::imm434/*URA*3Δ::imm434 *URA*3/*URA*3 MTLa/mtlα1::hisG mtlα2::hisG
2.	DSY2906	*TAC1*Δ::hisG/*TAC1*Δ::hisG LEU2::*TAC1*/*URA3*
3.	DSY2925	*TAC*1Δ::hisG/*TAC1*Δ::hisG LEU2::*TAC*1-*2*/*URA3*
4.	DSY2926	ZNC3/znc3Δ::hisG-*URA3*-hisG

**Figure 5:**
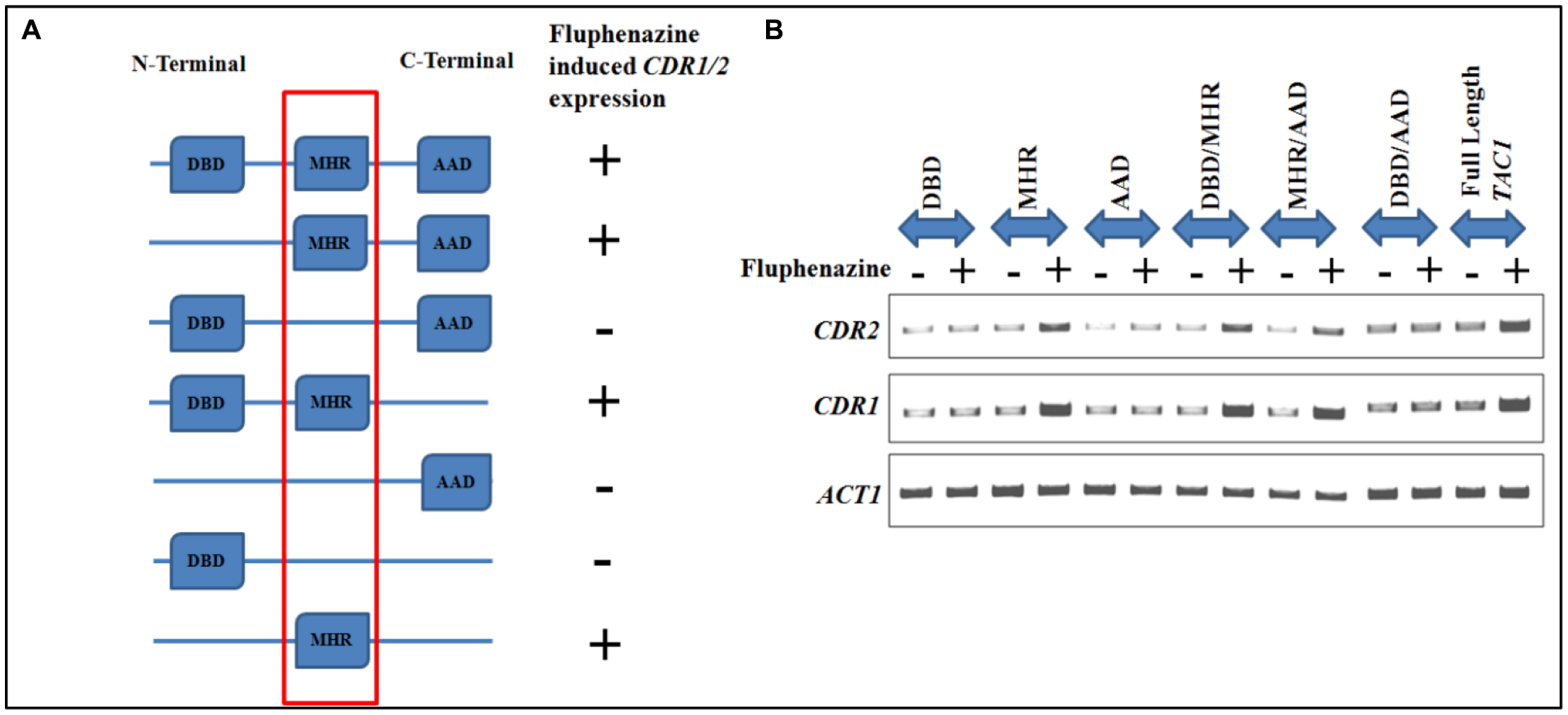
Domain dissection studies of *TAC1*. **(A)** Represents different domain combinations of *TAC1* that were cloned in the pVT50 *Candida* expression vector. All the domain combinations were transformed in the *TAC1*Δ strain of *C. albicans* DSY2906. Fluphenazine-induced up-regulation of target genes *CDR1* and *CDR2* was observed in constructs harboring the MHR domain. **(B)** Semi-quantitative PCR was performed in the absence and presence of Fluphenazine with different domain combinations of Tac1p, to check the mRNA expression level of *CDR* genes.

Further, to identify the region of Tac1p responsible for xenobiotic binding, various combinations of domain-dissected and full-length *TAC1* were cloned into the shuttle vector pVT50. The constructs were transformed in *TAC1* deletion background in strain *DSY2906* and cultured to subsequently process for RNA isolation upon induction with Fluphenazine. Semi-Quantitative PCR was conducted to check the expression of *CDR1* and *CDR2* genes and results showed up-regulation of these genes only in the presence of the Tac1-MHR domain. Various domain combinations and respective induction of *CDR* gene expression upon Fluphenazine treatment are shown in Figure 5. From the domain combination-permutation studies, it became evident that DNA constructs harboring the MHR domain were the only ones able to respond to Fluphenazine induction. From these studies, it became clear that Tac1-MHR acts as the Xenobiotic Binding Domain (XBD) and is responsible for interaction with drugs to bring about transcriptional up-regulation of target genes. Interestingly, we observed that even the construct containing MHR alone could induce the expression of *CDR1* and *CDR2* (Figure 5). It will be interesting to study the mechanism of DNA interaction and induction of *CDR* genes by MHR in future studies.

### Role of *TAC1* in the regulation of lipid profiling genes and stress response genes

**Figure 6:**
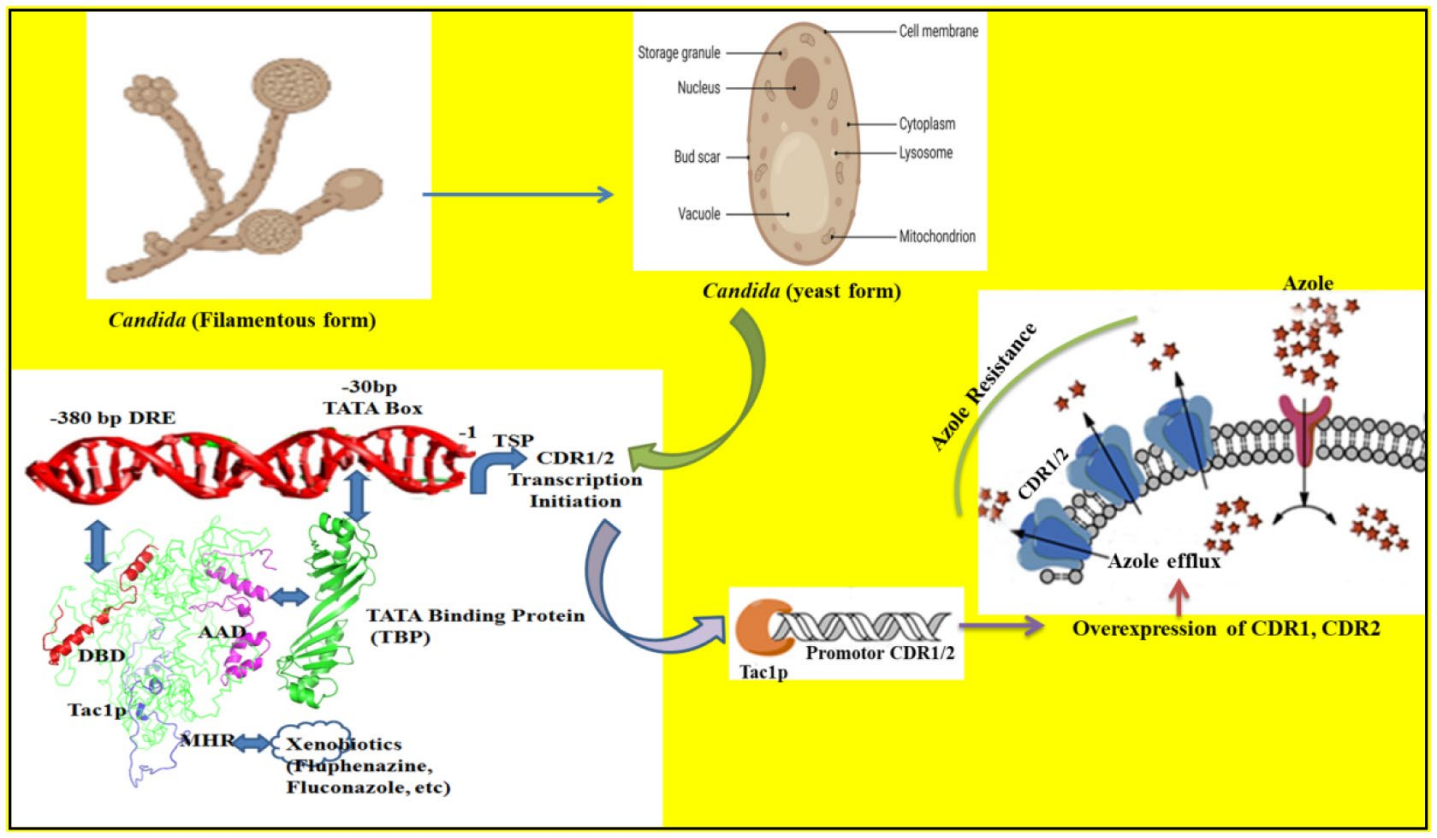
The binding of different domains of Tac1p and its influence on the transcription of *CDR1/2* genes is illustrated. The DBD domain binds with the −380 bp DRE sequence while the AAD domain binds with the TBP (Tata binding protein) and recruits it to the −30 bp TATA box. The MHR domain acts as the xenobiotic binding domain and helps Tac1p to respond to environmental cues.

Semi-quantitative PCR is an efficient tool to check the mRNA expression of desired genes. We checked the expression of lipid profiling genes as well as stress response genes to check whether Tac1p plays any role in the expression of these genes besides *CDR1* and *CDR2* genes. Among lipid profiling genes and stress response genes we have selected only those genes which have maximum similarity with 21 bases DRE sequences, our selected genes show 55 to 100% similarity with DRE sequence (Table 3). We checked gene expression in the presence and absence of xenobiotics (Fluphenazine) in the cultures of *C. albicans.* RNA was extracted from the grown *C. albicans* cells, and further, RNA was used to synthesize cDNA. Synthesized cDNA was used as a template for checking gene expression of target genes. Gene amplified Semi-quantitative PCR product was run onto the agarose gel and all the images were captured through Chemidoc LAS4000 (GE Healthcare) (Supplementary Figures S1A–H). All gel bands were quantified by using the Image quant gel bands analysis tool. The significance of the data was determined by using a one-way ANOVA statistical method. Among five tested lipid profiling genes, three (*ERG3*, *ERG6*, *ERG11*) showed a significantly increased level of gene expression in Fluphenazine-induced strains of *C. albicans* as compared to uninduced strains (Figure 6). Further, we also checked the expression of ten stress response genes and found significant changes in the expression of six genes *PDE2*, *SOD1*, *SOD5*, *SSK-1*, *TTR1*, and *UPC2,* especially in Fluphenazine-induced strains of *C. albicans* (Figure 4C).

**Table 3:** Multiple sequence alignment and percent similarity between consensus DRE sequence and promoter sequences of lipid profiling genes, stress response genes, and housekeeping genes of *Candida albicans*.

S.No.	Genes	Percent Identity	Sequence alignment between DRE and promoters of other genes
1	*CDR2*	100%	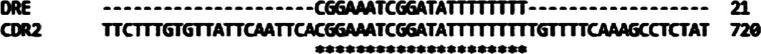
2	*CDR1*	95.24%	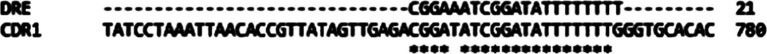
3	*ERG11*	71.43%	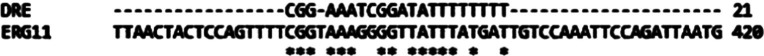
4	*SSK1*	71.43%	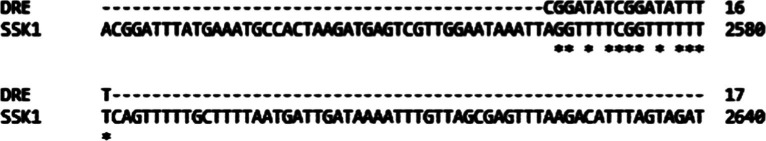
5	*TAC1*	71.43%	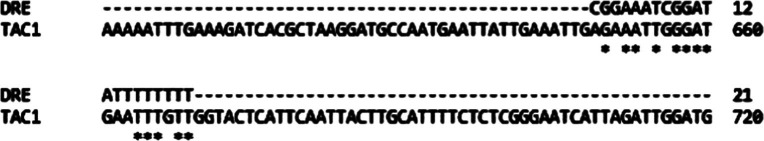
6	*Upc2*	66.67%	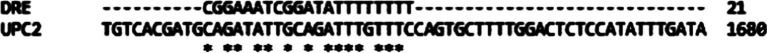
7	*ERG3*	66.67%	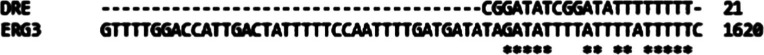
8	*ERG5*	66.67%	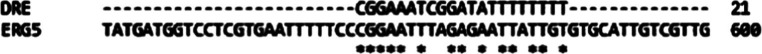
9	*TEF3*	66.67%	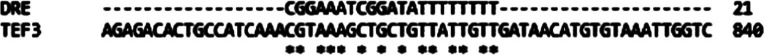
10	*CAP1*	66.67%	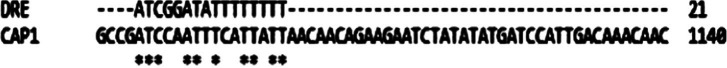
11	*SKN 7*	66.67%	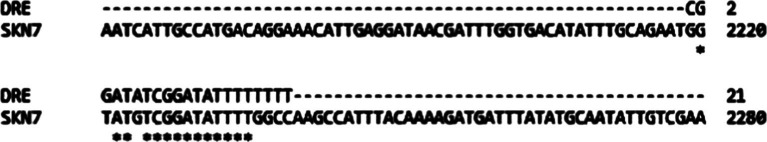
12	*PDE2*	66.67%	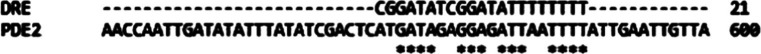
13	*HOG1*	61.9%	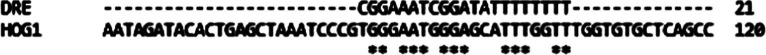
14	*NIK1*	61.9%	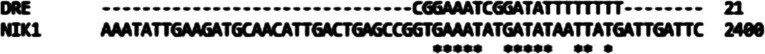
15	*TTR1*	61.9%	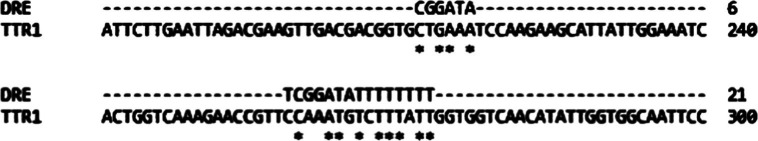
16	*SOD1*	61.9%	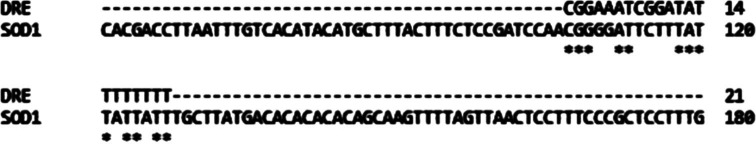
17	*SOD5*	61.9%	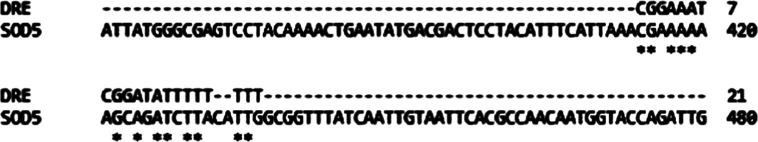
18	*ERG6*	57.14%	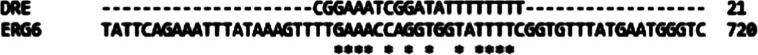
19	*ACT1*	57.14%	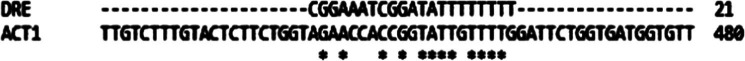

## Discussion

We conducted this study to characterize proteins involved in the Cdr1p transporter of *C. albicans* for the identification of novel targets for the development of targeted therapy against *Candida* spp. We investigated Tac1p binding to the DRE sequence of the *CDR* gene of *C. albicans*, via combined utilization of computational and wet-lab experimental approaches. In the absence of the X-ray crystal or solution NMR structures, theoretical models were generated and supported concomitantly with the biological experiments. The primary outcome of the *in silico* analysis yielded the most plausible architecture of the Tac1 DBD-DRE of *C. albicans,* which emerged as a homodimer with a direct repeat arrangement. A molecular dissection study identified the transcription factor TBP (TATA-binding protein) that interacts with Tac1-AAD and facilitates the formation of the pre-initiation complex to bring about transcription initiation of *CDR* genes. Eukaryotic transcription initiation requires several co-factors which act distantly from the transcription start site to facilitate the recruitment of RNA polymerase II (Hahn, 2004). Such co-factors can be trans-activators like Tac1p that bind to DRE (−380 bp) present in the *CDR* gene promoter region. The C-terminal AAD of Tac1p, much like Gal4-AD, brings about transcriptional initiation of reporter genes when present in proximity with the DNA binding domain (Gal4-BD), a principle on which Y2H assay works (Osman, 2004). We employed this principle to identify a putative co-factor *TBP,* which codes for TATA-box binding protein that mediates the interaction of Tac1-AAD with 30 bp upstream TATA-box in the promoter region of *CDR* genes. This interaction brings Tac1-DBD and Tac1-AAD in proximity to the promoter region of *CDR* genes to facilitate their transcription initiation. Functional studies further identified Tac1-MHR as Xenobiotic Binding Domain (XBD), which upon interaction with small molecules leads to up-regulation of *CDR* genes. However, events leading to up-regulation of target genes upon protein-ligand interaction must be studied in detail to shed more light on the structural aspect. There is a possibility that this interaction may lead to a change in conformation in the overall structural assembly of Tac1p on *CDR* promoter resulting in differential gene expression. In the near future, the role of other putative cofactors like *NCB2* may be identified (Shukla et al., 2011). Targeting of efflux pumps by agents with or without intrinsic anti-fungal activity was done in the past. Compounds such as ibuprofen (Ricardo et al., 2009), curcumin (Garcia-Gomes et al., 2012), FK506 (Uppuluri et al., 2008), and farnesol (Sharma and Prasad, 2011) were identified to directly inhibit ABC transporters in *C. albicans.* These molecules were able to chemo-sensitize *Saccharomyces cerevisiae* strains harboring *Candida* efflux pumps. However, considering the homology of ABC transporter proteins with human Pgp receptor, we may be better able to inhibit the efflux pumps indirectly, by targeting the transcriptional regulatory network. The transcription activator Tac1p belongs to Zn(2)Cys(6) class of Zn cluster proteins, which are specific to the fungal kingdom, thus providing a fungal-specific target to chemo-sensitize azole-resistant isolates of *Candida*.

The detailed molecular regulation of *CDR* genes via *TAC1* was investigated by performing molecular dissection studies, which revealed that the MHR domain is responsible for xenobiotic binding. This study presents the fungal-specific novel drug targets for the development of new-generation therapy for the treatment of candidiasis. In the coming future, we will try to find some potent therapeutic molecules that disrupt the Tac1 DBD-DRE interaction that can lead to the sensitization of azole-resistant strains of *C. albicans* to lower doses of azole drugs. Therefore, this study will be instrumental in drug discovery efforts targeting the Tac1p. In this study, we also tried to find out whether Tac1p regulates the expression of other genes. Among other genes, we have chosen lipid profiling genes (*UPC2*, *ERG3*, *ERG5*, *ERG6*, and *ERG11*) (Branco et al., 2017), stress response genes (*TAC1*, *CAP1*, *SKN7*, *HOG1*, *NIK1*, *SSK1*, *TTR1*, *PDE2*, *SOD1*, and *SOD5*) (Singh et al., 2020). We used *TEF3* and *ACT1* housekeeping genes as a control during entire gene expression studies. In this study, we evaluated gene expression in an uninduced and xenobiotic Fluphenazine-induced strain of *C. albicans*. For this study, we have used azole-sensitive strains (SC5314, GU4, and DSY294), azole-resistant strains (GU5 and DSY296), and Tac1 deleted strain DSY2906. Among all 17 evaluated genes, these are the following genes (*ERG6*, *PDE2*, *SKN7*, *SOD1*, *SOD5*, *TTR1*, *UPC2*, and *TAC1*) which showed statistically significant change in their expression. In most of the studied genes, their expression was increased after exposure to the Fluphenazine drug except the SOD5 gene which was downregulated in some strains of *C. albicans* especially in *TAC1* deleted strain DSY2906. As per the previous reports (Karababa et al., 2004; Costa-de-Oliveira and Rodrigues, 2020), *CDR1* and *CDR2* expression was increased after exposure to drugs due to the involvement of Tac1p in their promotor sites. Similarly, in all the above-mentioned genes, the presence of the 21 base pair DRE consensus sequence, which is the binding site of Tac1p protein, suggested that they may be regulated by Tac1p. Therefore, besides the *CDR1* and *CDR2* gene regulation, Tac1p also regulates the expression of other genes such as lipid profiling genes and stress response genes. In this study, we also confirmed that *TAC1* regulates its own expression because its promoter contains a DRE sequence with 71.43% sequence similarity with the DRE consensus sequence. Therefore, we concluded that Tac1p is a major factor needed not just for the regulation of *CDR1* and *CDR2*, but also other genes responsible for antifungal drug resistance in *C. albicans*.

## Summary

In summary, this study establishes the most plausible assembly of Tac1-DBD and DRE of *CDR1* of *C. albicans.* The AAD and MHR domains also have specific roles in xenobiotic signaling and recruitment of transcription machinery that leads to the upregulation of MDR genes like CDR1/2 (Figure 6). In the future, it will be our endeavor to try and disrupt the binding to Tac1p to DRE by novel small molecules that can target the Tac1p-DRE interaction (thereby inhibiting transcription factor binding) or by targeting the AAD-TBP interaction (thereby inhibiting the PPI mediated transcription).

## Materials and methods

### Media and chemicals

All the media components were procured from HiMedia (Mumbai, India). Antibiotics ampicillin (AMP) and kanamycin were purchased from Calbiochem, India. RNeasy mini kit for RNA isolation was bought from Qiagen, India. The reverse transcriptase kit was purchased from Thermo Scientific, India. dNTP mix, Fluconazole (Flu), Anti-GST, and Anti-His antibodies were purchased from Sigma Chemicals Co. (St. Louis, MO). Protino Ni-NTA Agarose resins were purchased from Macherey-Nagel, India, and Glutathione Sepharose 4B resin was purchased from Cytiva.

### Strains, plasmid, and culture conditions

*Escherichia coli* strain DH5α (Invitrogen) was used to amplify plasmids. *E. coli* strain BL21 (DE3) (Invitrogen) was used for the expression of recombinant proteins. The *C. albicans* strains used in this study were azole-sensitive strains SC5314, GU4, DSY294; clinical azole-resistant isolates GU5 and DSY296; Parent strain CAF4-2; Tac1 deletion strain DSY2906; Tac1 wild type revertant strain DSY2925; and Tac1 Gain of Function (GOF) revertant strain DSY2926 [10]. *Saccharomyces cerevisiae* strain PJ69-4A was used for Y2H studies (Uetz et al., 2000). Plasmid pET-28a and pGEX-KG (Invitrogen) were used for cloning and gene expression in *E. coli*. Shuttle vector pVT50 was used for cloning full-length *TAC1* and various domain combinations. Yeast-2-Hybrid (Y2H) studies were done using vectors pAS2 and pACT2 with Gal4-DNA Binding Domain (Gal4-DBD) and Activation Domain (AD) respectively. *E. coli* strains were maintained as glycerol stocks at −80°C. Bacteria were grown in Luria-Bertani (LB) broth or on LB agar plates (HiMedia, India) at 37°C in a shaker incubator (New Brunswick Scientific). Fungal strains were maintained as glycerol stock at −80°C and grown in complete medium YPD (Yeast Extract Peptone Dextrose) broth or YPD agar plates (HiMedia, India).

### PCR and cloning

*TAC1* DNA and protein sequences were obtained from the *Candida* genome database.^1^ The genomic DNA of *C. albicans* SC5314 was used to PCR amplify genes of interest. The DNA sequence encoding the DNA Binding Domain (33–80 AA) and Acidic Activation Domain (880–981 AA) was PCR amplified using gene-specific primers and cloned in pET28a and pGEX-KG expression vectors, respectively, with affinity tags. *C. albicans TBP1* was PCR amplified and cloned in the pET28a expression vector. Full-length *TAC1* and various domain combinations including DBD, MHR, AAD, DBD-MHR, MHR-AAD, and DBD-AAD were cloned in a pVT50 shuttle vector. AAD and *TBP1* DNA fragments were cloned in yeast expression vectors pACT2 and pAS2, respectively, for Y2H studies. Cloned sequences were confirmed by DNA sequencing. The clones and primers used in the study are listed in Table 2, Supplementary Table S1, and Supplementary Figure S2.

### Expression and purification of recombinant protein

For over-expression of recombinant proteins DBD, AAD, and TBP; the plasmid clones were transformed into *E. coli* expression host BL21 (DE3). Different induction conditions and media were tested for overexpression of recombinant proteins. For over-expression of DBD protein, cells were grown at 37°C in LB media to an OD_595_ 0.5, induced by the addition of 0.5 mM IPTG and 100 μM Zn(OAc)_2_ and grown for an additional 3 h. For AAD protein overexpression, cells were grown at 37°C in Terrific Broth (TB) media to an OD_595_ 0.5, induced by the addition of 1 mM IPTG and grown overnight at 16°C. TBP protein was over-expressed by culturing cells at 37°C in LB media to an OD_595_ 0.5 and induced by the addition of 1 mM IPTG followed by 6 h growth in a shaker incubator. After proteins were over-expressed, cells were pelleted down. The cells were lysed by sonication in a low salt buffer (LSB) containing 20 mM HEPES (pH 7.5), 200 mM NaCl, 1 mM β-ME, 100 mg/mL phenylmethylsulfonylfluoride (PMSF)/benzamidine, centrifuged, and the supernatant was loaded onto either Ni-NTA Agarose resin column or Glutathione Sepharose 4B resin column depending on affinity Tags of respective proteins. Affinity Tag purification was performed according to the manufacturer’s protocol. Pure protein fractions were pooled and dialyzed against LSB.

### Sequence analysis and homology modeling of Tac1-DBD domain

Homology modeling was based on the multiple 3D templates selected from the BLAST (Basic Local Alignment Search Tool) search of the FASTA sequence of Tac1-DBD domain (Swiss-Prot database id: A7IZW6) based on their low E-value, broadest query coverage, and high sequence similarity. Multiple sequence alignment was performed by ClustalW (Larkin et al., 2007). Cadmium from PDB ID 1D66 and 1CLD was manually modified to zinc atom and missing residues in the DBD domain region were added using Modeler 9.13 [18]. Modified template structures were initially selected based on the MOLPDF score and further refined through the steepest descent gradient minimization utilizing the CHARMM27 force field in Gromacs 4.6 package (Abraham et al., 2015). Homology modeling was carried out using Modeler 9.13 with two zinc atoms retained as such. The top 10 models were initially selected from 100 generated models based on DOPE, MOLPDF, and GA341 scores, and energy minimizations were performed with the CHARMM27 force field as mentioned above. The quality of the constructed models was determined with the Ramachandran plot [21], ProSA plot [22], and ERRAT plot [23], and the secondary structure were assigned with STRIDE [24]. The top model was selected based on the affirmative results from these analyses. Further, the coordination of two zinc atoms with six cysteine residues was optimized using a metal geometry module in UCSF Chimera 1.8.1 (Pettersen et al., 2004).

### DBD-DRE complex modeling

3D-DART [26] was used for DRE modeling with sequence input (5′-CGGATATCGGATATTTTTTTT-3′) and default parameters. High Ambiguity Driven biomolecular DOCKing (HADDOCK) was used for Tac1-DBD and DRE docking (performed on the WeNMR portal) [25]. Four different combinations of DRE base pairs (bp) *viz.* DRE1 [± 10 to ±7 (CGG)], DRE2 [± 3 to ±1 (CGG)], DRE3 [± 10 to ±7 (CGG) - ± 3 to ±1 (CGG)] and DRE4 [± 10 to ±1 (CGGATATCGG)] were assigned as restraints for docking and those nucleotides lying within 6.5 Å were automatically assigned as the passive residues. Full sequences of DBD were considered as active residues. A root mean square deviation (RMSD) cutoff of 7.5 Å for clustering with a minimum cluster size of four was selected as the default parameter. The RMSD is defined as the spatial difference between two static structures. RMSD is frequently applied as a numerical indicator of similarity between two or more protein structures. The smaller RMSD values indicate a better-suited model for the target structure. Values for RMSD are shown in Å (Kufareva and Abagyan, 2012). Final docked complexes were selected based on the highest cluster size, *z*-score, and visual inspections.

### Molecular dynamics simulation

Molecular dynamics (MD) simulation was performed in Gromacs 4.6 package with CHARMM27 [20] all-atom force field embedded in a truncated octahedron box of appropriate dimensions with periodic boundary conditions and TIP3P solvation model. The individual system was neutralized using appropriately adding the Na^+^ and Cl^−^ ions to the solution. SHAKE algorithm was used to constrain all bond lengths involving hydrogen atoms and long-range electrostatic interactions were treated with the Particle Mesh Ewald method (cutoff 12 Å). Systems were suitably minimized and then equilibrated in NPT and NVT ensemble to reach 1 atmospheric pressure and 300 K temperature. Production runs were carried out with a Parrinello−Rahman barostat to keep the system at constant pressure (1 atm) and temperature (300 K). Trajectories were collected at every 2.0 fs.

### GST pull-down assay

The recombinant AAD domain of Tac1p was purified using GST-sepharose resins and TBP was purified using Ni-NTA affinity chromatography according to the manufacturer’s protocol and dialyzed against LSB. Glutathione Sepharose 4B beads were incubated with recombinant AAD and TBP protein in binding buffer [20 mM HEPES, pH 7.5, 200 mM NaCl, 0.1 mM EDTA, 10% glycerol, 0.1%NP-40, 1 mM β-ME, 100 mg/mL phenylmethylsulfonyl fluoride (PMSF)/benzamidine] for 3 h at 4°C. As a control, purified GST and TBP proteins were incubated with Glutathione Sepharose 4B beads alone under similar conditions. The beads were washed three times with wash buffer [20 mM HEPES, pH 7.5, 500 mM NaCl, 0.1 mM EDTA, 10% Glycerol, 0.1% NP-40, 1 mM β-ME, 100 mg/mL phenylmethylsulfonyl fluoride (PMSF)/benzamidine] and once with binding buffer. The bound proteins were eluted in a binding buffer containing 10 mM reduced glutathione. The eluted proteins were resolved on 12% polyacrylamide gel and detected by immunoblotting.

### Yeast-2-hybrid assay

For yeast two-hybrid interaction studies, AAD and TBP were PCR amplified and cloned in pACT2 and pAS2 vectors respectively, using gene-specific primers. The plasmid containing AAD as prey was cloned in fusion with the GAL4 activation domain in vector pACT2. TBP protein was cloned in fusion with the DNA-binding domain of the pAS2 vector. AAD-pACT2 containing activation domain and TBP-pAS2 containing DNA-binding domain were co-transformed in the PJ69-4A strain (Clontech). Interaction studies were performed using LacZ and HIS3 as reporter genes on SD-leu-trp plates containing X-gal or lacking histidine, respectively (Van Criekinge and Beyaert, 1999).

### Yeast transformation

*Saccharomyces cerevisiae* strain PJ69-4a and *C. albicans TAC1* deletion strain DSY2906 were transformed using lithium acetate as described previously with slight modification (Pettersen et al., 2004). Briefly, yeast cells were grown overnight in 10 mL YPD broth. Cells were pellet down, washed twice with distilled water, and suspended in 0.1 mL solution containing 200 mM lithium acetate, 40% w/v polyethylene glycol (PEG), and 250 ng of denatured salmon sperm DNA. Transforming vectors (1 to 5 μg) were added to the yeast suspension followed by heat shock at 42°C for 10 min. Cells were then pelleted down and washed with distilled water to remove PEG, re-suspended in 100 μL distilled water, and directly plated onto agar plates containing selection media.

### RNA isolation and semi-quantitative PCR analysis of Tac1p-regulated genes

Each *C. albicans* strain was grown overnight in 10 mL of YPD broth at 30°C with constant agitation. For RNA extractions, cultures were diluted at 10^7^ cells per ml in 10 mL of fresh YEPD medium, grown at 30°C with constant agitation for 12 h then pelleted for RNA isolation using RNeasy mini kit (Qiagen, India), according to manufacturers’ instructions. For studying the effect of Fluphenazine induction on *CDR1* and *CDR2* genes, *TAC1* genes, lipid profiling genes (*ERG 3*, *ERG 5*, *ERG 6*, *ERG 11*, *UPC 2*) and stress response genes (*SSK1*, *CAP-1*, *SKN 7*, *PDE 2*, *HOG1*, *SOD 1*, *SOD 5*) expression. All these genes were selected based on their promoter sequence similarity with the DRE consensus sequence found in the promoter region of *CDR1* and *CDR2* genes of *C. albicans*. As per previous reports, Tac1p regulates the expression of several genes by recognizing DRE consensus sequences found in the promoter region of genes (Coste et al., 2004, 2006). Tac1p is a major regulator of CDR1 and CDR2 genes beside that we want to check whether Tac1p regulates the expression of lipid profiling genes and stress response genes (Sanglard et al., 2009). To validate the expression of these genes first, we checked promoter sequence similarity with the DRE sequence that is listed in Table 3. *C. albicans* cells were treated with 10 μg/mL of Fluphenazine (Fluphenazine dihydrochloride, MP Biomedicals), 15 min prior to processing culture for RNA isolation. cDNA synthesis was done using the Verso cDNA synthesis kit (Thermo Scientific). Briefly, 1 μg isolated RNA was primed with oligo dT for cDNA synthesis at 42°C for 60 min. The reverse transcription reaction was terminated by heating at 90°C for 2 min. The synthesized cDNA product (2 μL) was directly used for PCR amplification reaction (50 μL) using gene-specific forward and reverse primers listed in Table 4. The amplified products were separated by gel electrophoresis and quantitated and imaged using Image Quant LAS4000 (GE Healthcare) (Table 5).

**Table 4:** List of clones and primers used in this study.

S.No	Clones	Primer	Restriction site
1	DBD-pET28a	F Primer: 5-GATCAAGCTTTTCGAAACTCCGTTGCTATTGG-3 R Primer: 5-CATAAGCTTTCATTTCAATTTCTTTCTCAC-3	NdeI HindIII
2	TBP-pET28a	F Primer: 5-CATGAATTCATGGATTTAAAATTACCCCCA-3 R Primer: 5-CATAAGCTTTCAATTTTTACGAAATTCATTTAA-3	NdeI HindIII
3	AAD-pGEX-KG	F Primer: 5-CATGAATTCTACAAAATAACATGAACCCC -3 R Primer: 5-GATCAAGCTTAATCCCCAAATTATTGTCAAAG-3	EcoRI HindIII
4	*TAC1*-pVT50	F Primer: 5-AAACTCGAGATGGACACTTCACTGTCAC-3 R Primer: 5- GATCAAGCTTAATCCCCAAATTATTGTCAAAG −3	XhoI HindIII
5	DBD-pVT50	F Primer: 5-CAGCTCGAGATGGACACTTCACTGTCACTG-3 R Primer: 5-CAGAAGCTTTTAGTATTTCCTTTTCGAAACTCC GTTG −3	XhoI HindIII
6	MHR-pVT50	F Primer: 5-CAGCTCGAGATGGGTCTAGAAACAGTAGAAGCA TTG-3 R Primer: 5-CAGAAGCTTTTACAAAATATCTAAACAAATAGAG TCAGATCC-3	XhoI HindIII
7	AAD-pVT50	F Primer: 5-CAGCTGCAGATGGGTTGTGGTAGCAGGAACACC -3 R Primer: 5-TGCAAGCTTTTAAATCCCCAAATTATTGTCAAAG −3	PstI HindIII
8	DBD-MHR-pVT50	F Primer: 5-CAGCTCGAGATGGACACTTCACTGTCACTG-3 R Primer: 5-CAGAAGCTTTTACAAAATATCTAAACAAATAGA GTCAGATCC-3	XhoI HindIII
9	MHR-AAD-pVT50	F Primer: 5-CAGCTCGAGATGGGTCTAGAAACAGTAGAAGC ATTG-3 R Primer: 5-TGCAAGCTTTTAAATCCCCAAATTATTGTCAAAG −3	NcoI HindIII
10	DBD-AAD-pVT50	F Primer: 5-CAGCTCGAGATGGACACTTCACTGTCACTG-3 R Primer: 5-TGCAAGCTTTTAAATCCCCAAATTATTGTCAAAG −3	NcoI HindIII
11	TBP-pAS2	F Primer: 5-TGGCCATGGCCGATTTAAAATTACCCCCAACTA −3 R Primer: 5-GACGGATCCTCAATTTTTACGAAATTCATTTA −3	NcoI BamHI
12	AAD-pACT2	F Primer: 5-CCAAGCTTATGCCTCCAAAAAAGAAGAGAAAGG TCGAACAAAATAACATGAACCCCTCT-3 R Primer: 5-CCAAAGCTTCTAGCTAGCGTAATCTGGAACATCG TATGGGTAAGCAATCCCCAAATTATTGTCAAA-3	HindIII HindIII

**Table 5:** List of Primers used for semi-quantitative PCR of *Candida albicans* genes.

S.No.	Gene	Primer	Primer length	Tm (Salt adjustment)	GC Content	Product length
1.	*CDR1*	FP:5-TGCCAAACAATCCAACAA-3	18	52.82	38.89	112
		RP:5-CGACGGATCACCTTTCATACG-3	21	58.88	52.38	
2.	*CDR2*	FP:5-AAGGTTTTGATGCTACTG-3	18	49.70	38.89	125
		RP: 5-GTCGGACATGTGGCTCAAA-3	19	58.07	52.63	
1.	*Upc2*	F:-CCAGCACTTTTGGACAAGCAATTTATG	27	52	40.74	185
		R:-GCTCCACCTGCGTACTCTTC	20	51	60.00	
2.	*ERG3*	F:- CCAATCCAGTTGATGGGTTCTTCC	24	52	50.00	180
		R:- CAGTGTGACAAGCGGTACCATTG	23	52	52.17	
3.	*ERG5*	F:- GAAGAGCAATTGCGTGTGAGAAAC	24	51	45.83	226
		R:- CAGGGTGCAAAGCAGGATACAATG	24	52	50.00	
4.	*ERG6*	F:- GTGGTGTAGGTGGTCCTGGTAG	22	53	59.09	205
		R:-GCTTCAATGGCATAAACAGCATCG	24	51	45.83	
5.	*ERG11*	F:CTCATGGGGTTGCCAATGTTATGAAAAC	28	53	42.86	217
		R:- GAGATTTTCTTTTGAGCAGCATCACG	26	51	42.31	
6.	*CAP1*	F:- GAAGCATGTGGAACCAAAAGTAACC	25	51	44.00	176
		R:- CCCACACCATTGAAAAATGGATCATTC	27	52	40.74	
7.	*SKN 7*	F:- CCAAACTCAACACTTGCGACAACG	24	52	50.00	211
		R:- CAAAACTGGCAAAGTTTGAGTGCTTG	26	51	42.31	
8.	*HOG1*	F:- CACGTTGAACCGGAGGCTATTGA	23	52	52.17	247
		R:- CATTTGCCACACCAACAGTTTGATG	25	51	44.00	
9.	*NIK1*	F:- CCACGGTTATCACCAATGCAGC	22	52	54.55	184
		R:- CTTGTTGGAACGCTTTGTTGGCAT	24	51	45.83	
10.	*SSK1*	F:- GAACAACAAACTGCCGAACAGTCTG	25	53	48.00	186
		R:- GGATTGACTTGGCTTTCCTTTGCT	24	51	45.83	
11.	*TTR-1*	F:- CCAGTTTTCATTGCCTCCAAATCC	24	51	45.83	171
		R:- GACATTTGGAACGGTTCTTTGACC	24	51	45.83	
12.	*PDE2*	F:- GGGTTATTGGTTGCAGCATTGGG	23	52	52.17	247
		R:- CGGTGGCCAATATCGAAGAAATTATC	26	51	42.31	
13.	*SOD1*	F:- GTAAACAACATGGTGCTCCAGAAG	24	51	45.83	237
		R:- CACAAGCAGGTCTAGCACCAG	21	51	57.14	
14.	*SOD5*	F:- CTGACTCCAAAGGCAGTCCATC	22	52	54.55	247
		R:- GCAGCTCTAACGGTTCCATTGTAAG	25	53	48.00	
15.	*SEF3 or TEF3*	F:- GCCAGAAACCGTCCACTTGTTGG	23	54	56.52	249
		R:- GCTTCTGGATCAGCCATGTTGG	22	52	54.55	
16.	*ACT1*	F:- AAGAATTGATTTGGCTGGTAGAGA	24	58.18	37.50	179
		R:- TGGCAGAAGATTGAGAAGAAGTTT	24	58.62	37.50	
17.	*TAC1*	F:- TCCCGAGAGAAAATGCAAGT	29	54.53	20.69	182
		R:- TCACTATCGCCCACTCCTTC	27	58.58	33.33	

### Primer designing

Before primer design, we shortlisted lipid profiling genes and stress response genes based on a literature survey (Lv et al., 2016) of *C. albicans*, which may be regulated by the Tac1p protein of *C. albicans*. For primer designing, first, we acquired the FASTA coding DNA sequence of all the desired genes, from the Candida Genome Database (CGD) (Candida Genome Database, n.d.), then designed primer by putting FASTA DNA sequences in Primer3 web version 4.1.0 online software (Primer3 Input, n.d.). A list of designed primers and their characteristic features is attached in Table 2.

## Data availability statement

The raw data supporting the conclusions of this article will be made available by the authors, without undue reservation.

## Author contributions

DB designed the manuscript, got funds for working on the manuscript, and reviewed and wrote parts of the manuscript. TJ and SK performed the biological experiments. PM performed the modeling and *in silico* experiments. GP oversaw the *in silico* experiments and reviewed the manuscript. All authors contributed to the article and approved the submitted version.

## Funding

Financial support for this research was provided by the Council of Scientific and Industrial Research (CSIR)-India (Grant BSC0001 and MLP019) and Department of Science and 607 Technology (DST) grant from Govt of India, (Grant GAP0344).

## Conflict of interest

The authors declare that the research was conducted in the absence of any commercial or financial relationships that could be construed as a potential conflict of interest.

## Publisher’s note

All claims expressed in this article are solely those of the authors and do not necessarily represent those of their affiliated organizations, or those of the publisher, the editors and the reviewers. Any product that may be evaluated in this article, or claim that may be made by its manufacturer, is not guaranteed or endorsed by the publisher.

## Footnotes

^1^www.candidagenomedatabase.org

## Abbreviations

MDR: Multidrug Resistance
ABC: ATP-binding cassette
CDR1/2: *Candida* drug resistance 1/2
DBD: DNA Binding Domain
DRE: Drug Responsive Element
XBD: Xenobiotic binding domain
AAD: Acidic Activation Domain
MHR: Middle Homology Region
PPI: Protein–protein interaction
COVID-19: Coronavirus disease of 2019

## References

[ref1] Abraham M. J. Murtola T. Schulz R. Páll S. Smith J. C. Hess B.. (2015). Gromacs: high-performance molecular simulations through multi-level parallelism from laptops to supercomputers. SoftwareX 1-2, 19–25. doi: 10.1016/j.softx.2015.06.001

[ref2] Akins R. A. Sobel J. D. (2017). Antifungal targets, Mechanisms of Drug Resistance, and resistance in *C. albicans*. Antimicrob. Drug Resist. 1, 429–475. doi: 10.1007/978-3-319-46718-4_30

[ref3] Branco J. Ola M. Silva R. M. Fonseca E. Gomes N. C. Martins-Cruz C.. (2017). Impact of ERG3 mutations and expression of ergosterol genes controlled by UPC2 and NDT80 in *Candida parapsilosis* azole resistance. Clin. Microbiol. Infect. 23, 575.e1–575.e8. doi: 10.1016/j.cmi.2017.02.002 28196695

[ref4] Candida Genome Database (n.d.). Candida Genome. Available at: http://www.candidagenome.org/ (Accessed November 23, 2022)

[ref5] Chen M. Lin X. Lu W. Schafer N. P. Onuchic J. N. Wolynes P. G. (2018). Template-guided protein structure prediction and refinement using optimized folding landscape force Fields. J. Chem. Theory Comput. 14, 6102–6116. doi: 10.1021/acs.jctc.8b00683, PMID: 30240202 PMC6713208

[ref6] Costa C. Dias P. J. SÃ¡-Correia I. Teixeira M. C. (2014). MFS multidrug transporters in pathogenic fungi: do they have real clinical impact? Front. Physiol. 5:197. doi: 10.3389/fphys.2014.00197, PMID: 24904431 PMC4035561

[ref7] Costa-de-oliveira S. Rodrigues A. G. (2020). *C. albicans* antifungal resistance and tolerance in bloodstream infections: the triad yeast-host-antifungal. Microorganisms 8:154. doi: 10.3390/microorganisms8020154, PMID: 31979032 PMC7074842

[ref8] Coste A. T. Karababa M. Ischer F. Bille J. Sanglard D. (2004). TAC1, transcriptional activator of CDR genes, is a new transcription factor involved in the regulation of *C. albicans* ABC transporters CDR1 and CDR2. Eukaryot. Cell 3, 1639–1652. doi: 10.1128/EC.3.6.1639-1652.2004, PMID: 15590837 PMC539021

[ref9] Coste A. Turner V. Ischer F. Morschhäuser J. Forche A. Selmecki A.. (2006). A mutation in Tac1p, a transcription factor regulating CDR1 and CDR2, is coupled with loss of heterozygosity at chromosome 5 to mediate antifungal resistance in *C. albicans*. Genetics 172, 2139–2156. doi: 10.1534/genetics.105.054767, PMID: 16452151 PMC1456413

[ref10] Cowen L. E. Sanglard D. Howard S. J. Rogers P. D. Perlin D. S. (2015). Mechanisms of antifungal drug resistance. Cold Spring Harb. Perspect. Med. 5:752. doi: 10.1101/cshperspect.a019752, PMID: 25384768 PMC4484955

[ref11] De Micheli M. Bille J. Schueller C. Sanglard D. (2002). A common drug-responsive element mediates the upregulation of the *C. albicans* ABC transporters CDR1 and CDR2, two genes involved in antifungal drug resistance. Mol. Microbiol. 43, 1197–1214. doi: 10.1046/j.1365-2958.2002.02814.x, PMID: 11918807

[ref12] De Vries S. J. Van Dijk M. Bonvin A. M. J. J. (2010). The HADDOCK web server for data-driven biomolecular docking. Nat. Protoc. 5, 883–897. doi: 10.1038/nprot.2010.32, PMID: 20431534

[ref13] Fling M. E. Kopf J. Tamarkin A. Gorman J. A. Smith H. A. Koltin Y. (1991). Analysis of a *C. albicans* gene that encodes a novel mechanism for resistance to benomyl and methotrexate. MGG Mol. Gen. Genet. 227, 318–329. doi: 10.1007/BF00259685, PMID: 2062311

[ref14] Garcia-Gomes A. S. Curvelo J. A. R. Soares R. M. A. Ferreira-Pereira A. (2012). Curcumin acts synergistically with fluconazole to sensitize a clinical isolate of *C. albicans* showing a MDR phenotype. Med. Mycol. 50, 26–32. doi: 10.3109/13693786.2011.578156 21539505

[ref15] Hahn S. (2004). Structure and mechanism of the RNA polymerase II transcription machinery. Nat. Struct. Mol. Biol. 11, 394–403. doi: 10.1038/nsmb763, PMID: 15114340 PMC1189732

[ref16] Hameed S. Fatima Z. (2013). Novel regulatory mechanisms of pathogenicity and virulence to combat MDR in *C. albicans*. Int. J. Microbiol. 2013, 1–10. doi: 10.1155/2013/240209, PMID: 24163696 PMC3791847

[ref17] Heinig M. Frishman D. (2004). STRIDE: a web server for secondary structure assignment from known atomic coordinates of proteins. Nucleic Acids Res. 32, W500–W502. doi: 10.1093/nar/gkh429, PMID: 15215436 PMC441567

[ref18] Hellauer K. Rochon M. H. Turcotte B. (1996). A novel DNA binding motif for yeast zinc cluster proteins: the Leu3p and Pdr3p transcriptional activators recognize everted repeats. Mol. Cell. Biol. 16, 6096–6102. doi: 10.1128/mcb.16.11.6096 8887639 PMC231612

[ref19] Holmes A. R. Lin Y. H. Niimi K. Lamping E. Keniya M. Niimi M.. (2008). ABC transporter Cdr1p contributes more than Cdr2p does to fluconazole efflux in fluconazole-resistant *C. albicans* clinical isolates. Antimicrob. Agents Chemother. 52, 3851–3862. doi: 10.1128/AAC.00463-08, PMID: 18710914 PMC2573144

[ref20] Hooft R. W. W. Sander C. Vriend G. (1997). Objectively judging the quality of a protein structure from a ramachandran plot. Bioinformatics 13, 425–430. doi: 10.1093/bioinformatics/13.4.425 9283757

[ref21] Karababa M. Coste A. T. Rognon B. Bille J. Sanglard D. (2004). Comparison of gene expression profiles of *C. albicans* azole-resistant clinical isolates and laboratory strains exposed to drugs inducing multidrug transporters. Antimicrob. Agents Chemother. 48, 3064–3079. doi: 10.1128/AAC.48.8.3064-3079.2004, PMID: 15273122 PMC478486

[ref22] Kofla G. Turner V. Schulz B. Storch U. Froelich D. Rognon B.. (2011). Doxorubicin induces drug efflux pumps in *C. albicans*. Med. Mycol. 49, 132–142. doi: 10.3109/13693786.2010.512022, PMID: 20818920

[ref23] Ksiezopolska E. Gabaldón T. (2018). Evolutionary emergence of drug resistance in candida opportunistic pathogens. Genes 9:461. doi: 10.3390/genes9090461 30235884 PMC6162425

[ref24] Kufareva I. Abagyan R. (2012). Methods of protein structure comparison. Methods Mol. Biol. 857, 231–257. doi: 10.1007/978-1-61779-588-6_10, PMID: 22323224 PMC4321859

[ref25] Kusch H. Biswas K. Schwanfelder S. Engelmann S. Rogers P. D. Hecker M.. (2004). A proteomic approach to understanding the development of multidrug-resistant *C. albicans* strains. Mol. Gen. Genomics. 271, 554–565. doi: 10.1007/s00438-004-0984-x, PMID: 15114480

[ref26] Lage H. (2003). ABC-transporters: implications on drug resistance from microorganisms to human cancers. Int. J. Antimicrob. Agents 22, 188–199. doi: 10.1016/S0924-8579(03)00203-6, PMID: 13678820

[ref27] Larkin M. A. Blackshields G. Brown N. P. Chenna R. McGettigan P. A. McWilliam H.. (2007). Clustal W and Clustal X version 2.0. Bioinformatics 23, 2947–2948. doi: 10.1093/bioinformatics/btm404, PMID: 17846036

[ref28] Liu T. T. Znaidi S. Barker K. S. Xu L. Homayouni R. Saidane S.. (2007). Genome-wide expression and location analyses of the *C. albicans* Tac1p regulon. Eukaryot. Cell 6, 2122–2138. doi: 10.1128/EC.00327-07 17905926 PMC2168409

[ref29] Lv Q. Z. Yan L. Jiang Y. Y. (2016). The synthesis, regulation, and functions of sterols in *C. albicans*: well-known but still lots to learn. Virulence 7, 649–659. doi: 10.1080/21505594.2016.1188236, PMID: 27221657 PMC4991322

[ref30] MacPherson S. Larochelle M. Turcotte B. (2006). A fungal family of transcriptional regulators: the zinc cluster proteins. Microbiol. Mol. Biol. Rev. 70, 583–604. doi: 10.1128/mmbr.00015-06, PMID: 16959962 PMC1594591

[ref31] Messaoudi A. Belguith H. Ben Hamida J. (2013). Homology modeling and virtual screening approaches to identify potent inhibitors of VEB-1 β-lactamase. Theor. Biol. Med. Model. 10:22. doi: 10.1186/1742-4682-10-22, PMID: 23547944 PMC3668210

[ref32] Osman A. (2004). Yeast two-hybrid assay for studying protein-protein interactions. Methods Mol. Biol. 270, 403–422. doi: 10.1385/1-59259-793-9:403 15153642

[ref33] Paul S. Moye-Rowley W. S. (2014). Multidrug resistance in fungi: regulation of transporter-encoding gene expression. Front. Physiol. 5:143. doi: 10.3389/fphys.2014.00143, PMID: 24795641 PMC3997011

[ref34] Pettersen E. F. Goddard T. D. Huang C. C. Couch G. S. Greenblatt D. M. Meng E. C.. (2004). UCSF chimera - a visualization system for exploratory research and analysis. J. Comput. Chem. 25, 1605–1612. doi: 10.1002/jcc.20084, PMID: 15264254

[ref35] Prasad R. Banerjee A. Khandelwal N. K. Dhamgaye S. (2015). The ABCs of *C. albicans* multidrug transporter Cdr1. Eukaryot. Cell 14, 1154–1164. doi: 10.1128/EC.00137-15, PMID: 26407965 PMC4664872

[ref36] Prasad R. Devaux F. Dhamgaye S. Banerjee D. (2012). Response of pathogenic and non-pathogenic yeasts to steroids. J. Steroid Biochem. Mol. Biol. 129, 61–69. doi: 10.1016/j.jsbmb.2010.11.011, PMID: 21115115

[ref37] Primer3 Input (n.d.). primer3. Available at: https://primer3.ut.ee/ (Accessed November 23, 2022).

[ref38] Ren B. Dai H. Q. Pei G. Tong Y. J. Zhuo Y. Yang N.. (2014). ABC transporters coupled with the elevated ergosterol contents contribute to the azole resistance and amphotericin B susceptibility. Appl. Microbiol. Biotechnol. 98, 2609–2616. doi: 10.1007/s00253-013-5425-5, PMID: 24435642

[ref39] Ricardo E. Costa-de-Oliveira S. Silva Dias A. Guerra J. Ã. Rodrigues A. Ã.¡. G. Ã.§. Pina-Vaz C. Ã.¡. (2009). Ibuprofen reverts antifungal resistance on *C. albicans* showing overexpression of CDR genes. FEMS Yeast Res. 9, 618–625. doi: 10.1111/j.1567-1364.2009.00504.x, PMID: 19416368

[ref40] Robbins N. Wright G. D. Cowen L. E. (2016). Antifungal drugs: the current armamentarium and development of new agents. Microbiol. Spectrum 4:2016. doi: 10.1128/microbiolspec.funk-0002-2016, PMID: 27763259

[ref41] Roemer T. Krysan D. J. (2014). Antifungal drug development: challenges, unmet clinical needs, and new approaches. Cold Spring Harb. Perspect. Med. 4:19703. doi: 10.1101/cshperspect.a019703, PMID: 24789878 PMC3996373

[ref42] Sanglard D. Coste A. Ferrari S. (2009). Antifungal drug resistance mechanisms in fungal pathogens from the perspective of transcriptional gene regulation. FEMS Yeast Res. 9, 1029–1050. doi: 10.1111/j.1567-1364.2009.00578.x, PMID: 19799636

[ref43] Sharma M. Prasad R. (2011). The quorum-sensing molecule farnesol is a modulator of drug efflux mediated by ABC multidrug transporters and synergizes with drugs in *C. albicans*. Antimicrob. Agents Chemother. 55, 4834–4843. doi: 10.1128/AAC.00344-11, PMID: 21768514 PMC3186959

[ref44] Shukla S. Yadav V. Mukhopadhyay G. Prasad R. (2011). Ncb2 is involved in activated transcription of CDR1 in azole-resistant clinical isolates of *C. albicans* ∇. Eukaryot. Cell 10, 1357–1366. doi: 10.1128/EC.05041-11, PMID: 21856931 PMC3187062

[ref45] Singh S. Rehman S. Fatima Z. Hameed S. (2020). Protein kinases as potential anticandidal drug targets. Front. Biosci. Landmark 25, 1412–1432. doi: 10.2741/4862, PMID: 32114439

[ref46] Tanwar J. das S. Fatima Z. Hameed S. (2014). Multidrug resistance: an emerging crisis. Interdiscipl. Persp. Infect. Dis. 2014, 1–7. doi: 10.1155/2014/541340, PMID: 25140175 PMC4124702

[ref47] UCSF Chimera Home Page. (n.d.). UCSF Chimera. Available at: https://www.cgl.ucsf.edu/chimera/ (Accessed November 23, 2022).

[ref48] Uetz P. Giot L. Cagney G. Mansfield T. A. Judson R. S. Knight J. R.. (2000). A comprehensive analysis of protein-protein interactions in *Saccharomyces cerevisiae*. Nature 403, 623–627. doi: 10.1038/35001009 10688190

[ref49] Uppuluri P. Nett J. Heitman J. Andes D. (2008). Synergistic effect of calcineurin inhibitors and fluconazole against *C. albicans* biofilms. Antimicrob. Agents Chemother. 52, 1127–1132. doi: 10.1128/AAC.01397-07, PMID: 18180354 PMC2258509

[ref50] Van Criekinge W. Beyaert R. (1999). Yeast two-hybrid: state of the art. Biol. Procedures 2, 1–38. doi: 10.1251/bpo16, PMID: 12734586 PMC140126

[ref51] van Dijk M. Bonvin A. M. J. J. (2009). 3D-DART: a DNA structure modelling server. Nucleic Acids Res. 37, W235–W239. doi: 10.1093/nar/gkp287, PMID: 19417072 PMC2703913

[ref52] Vanommeslaeghe K. Hatcher E. Acharya C. Kundu S. Zhong S. Shim J.. (2010). CHARMM general force field: a force field for drug-like molecules compatible with the CHARMM all-atom additive biological force fields. J. Comput. Chem. 31:690. doi: 10.1002/jcc.21367, PMID: 19575467 PMC2888302

[ref53] Webb B. Sali A. (2016). Comparative protein structure modeling using MODELLER. Curr. Protoc. Bioinformatics 54, 5.6.1–5.6.37. doi: 10.1002/cpbi.3, PMID: 27322406 PMC5031415

[ref54] Wiederstein M. Sippl M. J. (2007). ProSA-web: interactive web service for the recognition of errors in three-dimensional structures of proteins. Nucleic Acids Res. 35, W407–W410. doi: 10.1093/nar/gkm290, PMID: 17517781 PMC1933241

[ref55] Znaidi S. de Deken X. Weber S. Rigby T. Nantel A. Raymond M. (2007). The zinc cluster transcription factor Tac1p regulates PDR16 expression in *C. albicans*. Mol. Microbiol. 66, 440–452. doi: 10.1111/j.1365-2958.2007.05931.x, PMID: 17897373

