# Supplementary material for: Molecular dissection studies of TAC1, a transcription activator of Candida drug resistance genes of the human pathogenic fungus Candida albicans
Source: Front Microbiol. 2023 Jul 12;14:994873. doi: 10.3389/fmicb.2023.994873 (PMC10370356; doi:10.3389/fmicb.2023.994873)
Supplement: Supplementary file 1 [file Data_Sheet_1.pdf]

Molecular dissection studies of *TAC1,* a transcription activator of *CDR* genes of the human pathogenic fungus *Candida albicans*

Tushar Jain,[a,b*] Pankaj Mishra,[b*] Sushil Kumar,[b*] Gautam Panda,[a,b, #] Dibyendu Banerjee[a,b,#]

[a]Academy of Scientific and Innovative Research (AcSIR), Ghaziabad- 201002, India.

[b]CSIR-Central Drug Research Institute, Sector-10, Janakipuram Extension, Sitapur Road, Lucknow-226031, UP, India.

* Authors contributed equally

#Address correspondence to Dr. Dibyendu Banerjee, Ph.D., Principal Scientist, Biochemistry & Structural Biology and Cancer Biology Divisions, CSIR-Central Drug Research Institute, Lucknow-226 031, U.P., India. Phone: (+91)522-2772450 Extn. 4443; Fax: (+91)522-2771941/42; E.mail: d.banerjee@cdri.res.in.

Dr. Gautam Panda, Ph.D., Chief Scientist, Medicinal and Process Chemistry Division, CSIR-Central Drug Research Institute, Lucknow-226 031, U.P., India. Phone: (+91)522-2772450 Extn. 4661; Fax: (+91)522-2771941/42; E.mail: gautam_panda@cdri.res.in

Supplementary Figures & Tables

Content:

Figure S1(A): Agarose gel electrophoresis of amplified *ERG6* gene through semi-quantitative RT-PCR, in an uninduced and fluphenazine-induced azole sensitive as well as resistant strains of *Candida albicans*.

Figure S1(B): Agarose gel electrophoresis of amplified *PDE2* gene through semi-quantitative RT-PCR, in an uninduced and fluphenazine-induced azole sensitive as well as resistant strains of *Candida albicans*.

Figure S1(C): Agarose gel electrophoresis of amplified *SKN7* gene through semi-quantitative RT-PCR, in uninduced and fluphenazine-induced azole sensitive as well as resistant strains of *Candida albicans*.

Figure S1(D): Agarose gel electrophoresis of amplified *SOD1* gene through semi-quantitative RT-PCR, in an uninduced and fluphenazine-induced azole sensitive as well as resistant strains of *Candida albicans*.

Figure S1(E): Agarose gel electrophoresis of amplified *SOD5* gene through semi-quantitative RT-PCR, in an uninduced and fluphenazine-induced azole sensitive as well as resistant strains of *Candida albicans*.

Figure S1(F): Agarose gel electrophoresis of amplified *UPC2* gene through semi-quantitative RT-PCR, in an uninduced and fluphenazine induced azole sensitive as well as resistant strains of *Candida albicans*.

Figure S1(G): Agarose gel electrophoresis of amplified *TAC1* gene through semi-quantitative RT-PCR, in an uninduced and fluphenazine induced azole sensitive as well as resistant strains of *Candida albicans*.

Figure S1(H): Agarose gel electrophoresis of amplified *TEF3* housekeeping gene through semi-quantitative RT-PCR, in an uninduced and Fluphenazine induced azole sensitive as well as resistant strains of *Candida albicans*.

Table S(1): Primers for cloning of MHR domain of Tac1p.

Figure S(2): Cloning of MHR domain in pET28a plasmid vector (A) Agarose gel electrophoresis of pET28a plasmid vector digested with BamHI & HindIII restriction enzymes. (B) Gel electrophoresis of the PCR product of MHR digested with BamHI & HindIII restriction enzymes. (C) Gel electrophoresis of clone confirmed with double restriction digestion with BamHI & HindIII restriction enzymes.

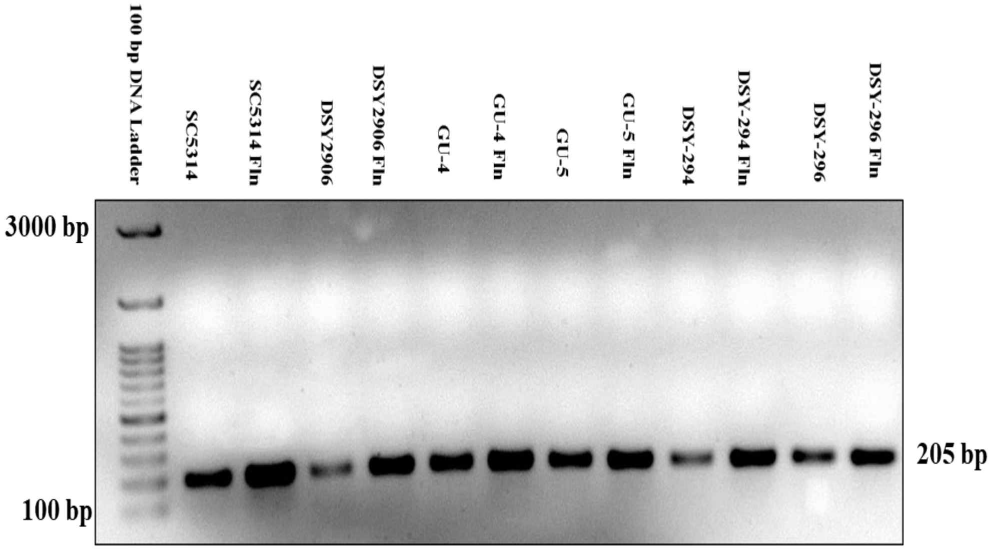

Figure S1(A): Agarose gel electrophoresis of amplified *ERG6* gene through semi-quantitative RT-PCR, in an uninduced and fluphenazine-induced azole sensitive as well as resistant strains of *Candida albicans*.

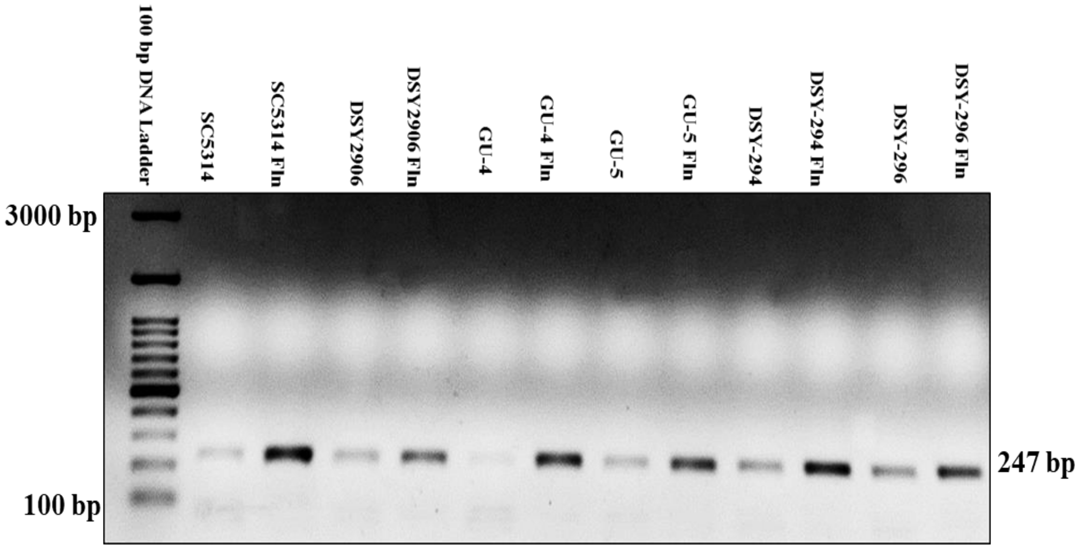

Figure S1(B): Agarose gel electrophoresis of amplified *PDE2* gene through semi-quantitative RT-PCR, in an uninduced and fluphenazine-induced azole sensitive as well as resistant strains of *Candida albicans*.

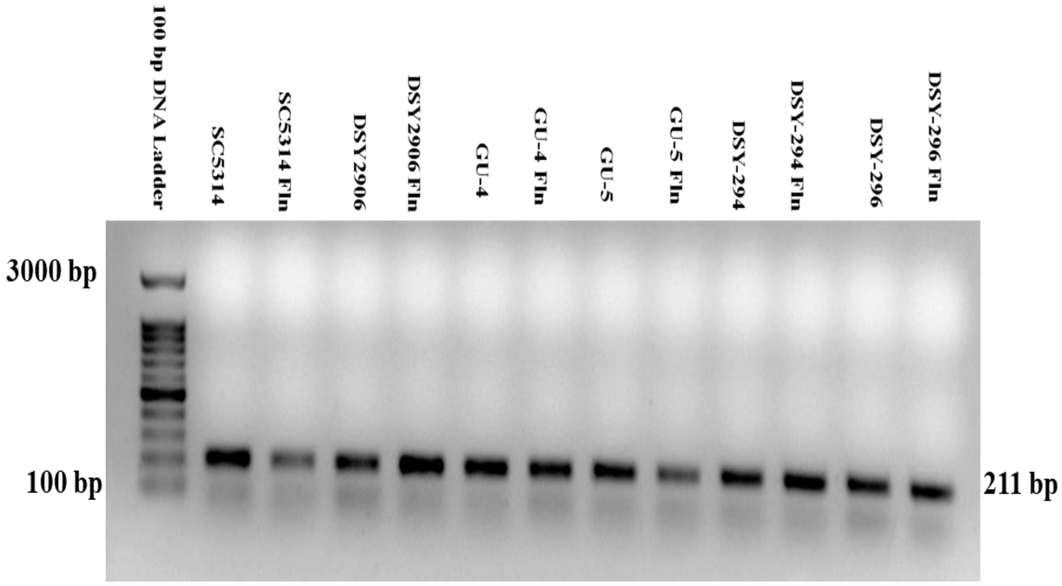

Figure S1(C): Agarose gel electrophoresis of amplified *SKN7* gene through semi-quantitative RT-PCR, in uninduced and fluphenazine-induced azole sensitive as well as resistant strains of *Candida albicans*.

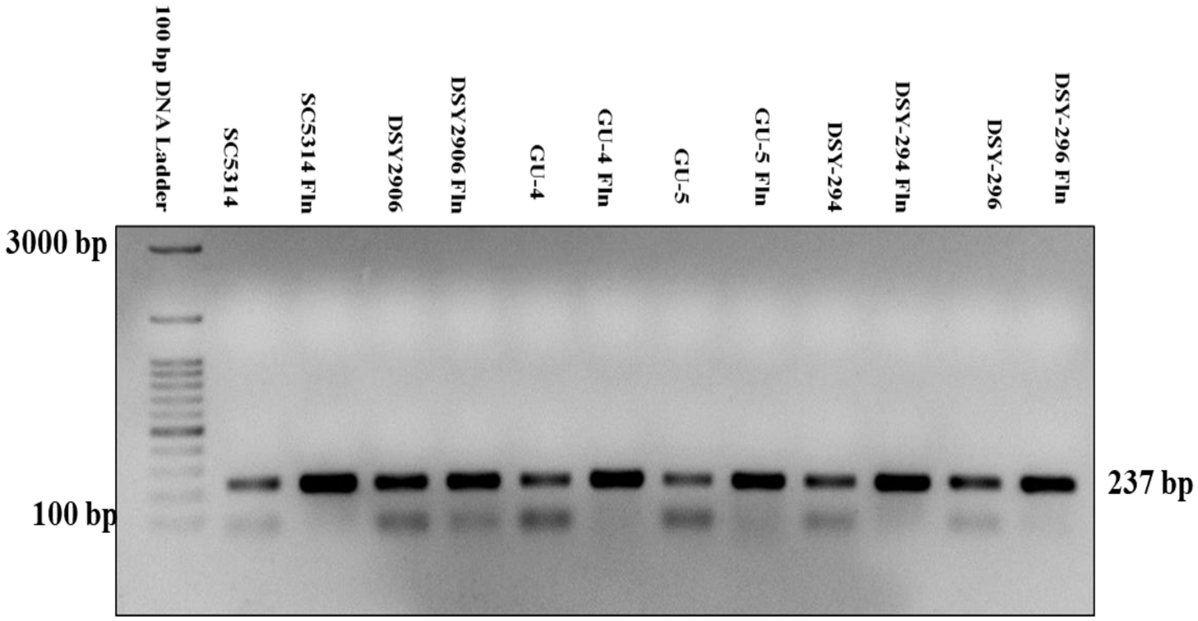

Figure S1(D): Agarose gel electrophoresis of amplified *SOD1* gene through semi-quantitative RT-PCR, in an uninduced and fluphenazine-induced azole sensitive as well as resistant strains of *Candida albicans*.

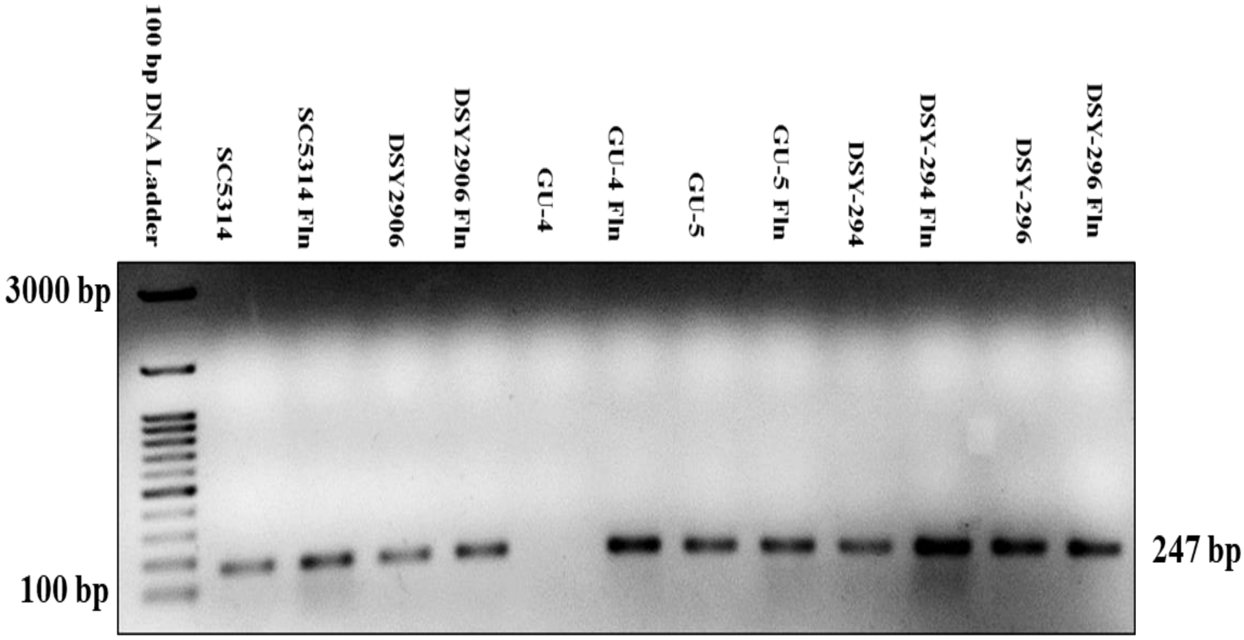

Figure S1(E): Agarose gel electrophoresis of amplified *SOD5* gene through semi-quantitative RT-PCR, in an uninduced and fluphenazine-induced azole sensitive as well as resistant strains of *Candida albicans*.

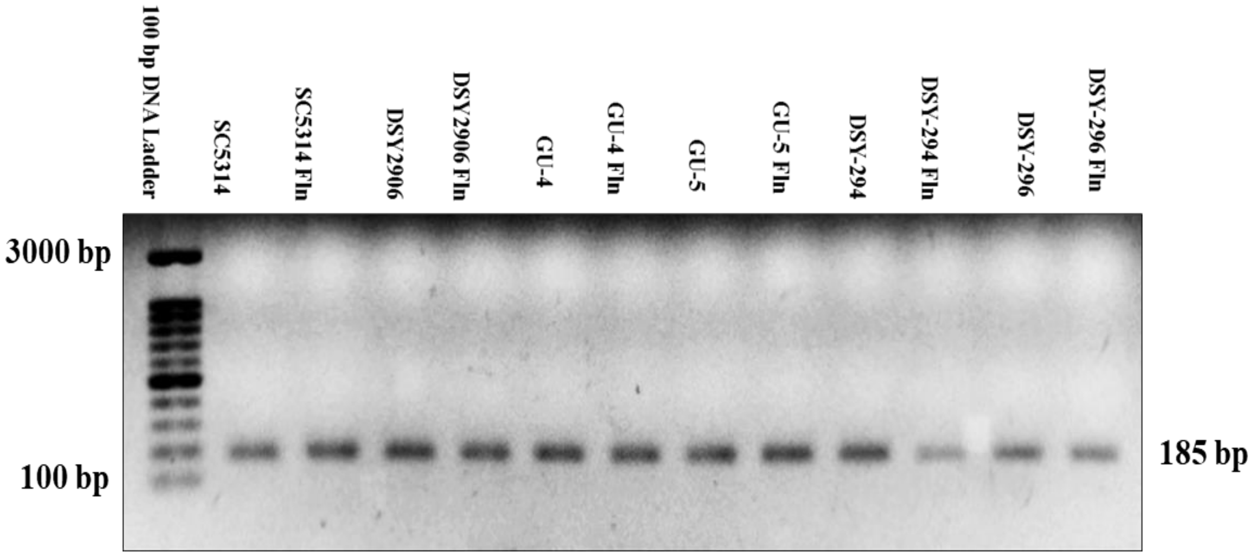

Figure S1(F): Agarose gel electrophoresis of amplified *UPC2* gene through semi-quantitative RT-PCR, in an uninduced and fluphenazine induced azole sensitive as well as resistant strains of *Candida albicans*.

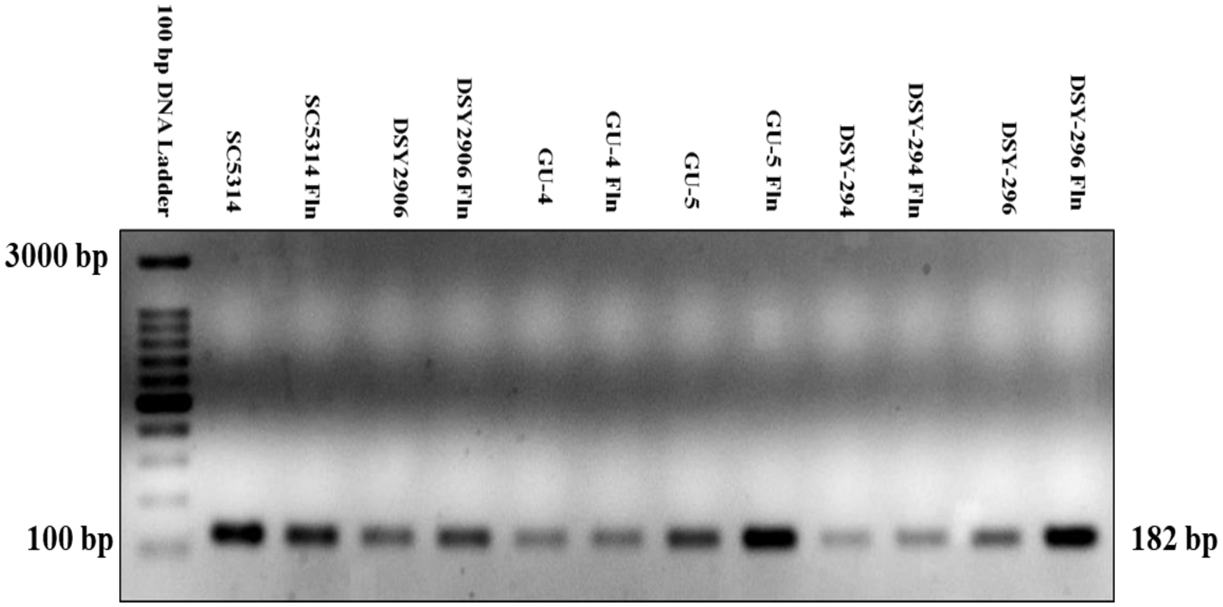

Figure S1(G): Agarose gel electrophoresis of amplified *TAC1* gene through semi-quantitative RT-PCR, in an uninduced and fluphenazine induced azole sensitive as well as resistant strains of *Candida albicans*.

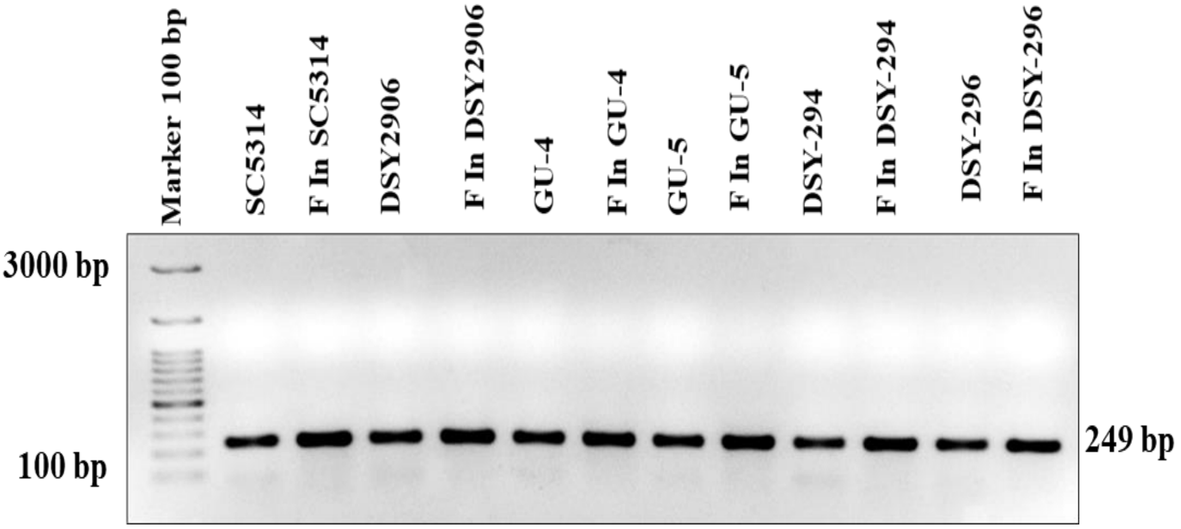

Figure S1(H): Agarose gel electrophoresis of amplified *TEF3* housekeeping gene through semi-quantitative RT-PCR, in an uninduced and Fluphenazine induced azole sensitive as well as resistant strains of *Candida albicans*.

Table S(1): Primers for cloning of MHR domain of Tac1p.

S.No.	Primers	Primer sequence	Primer Length	Restriction sites
1.	MHR Forward Primer	5' AAAGGATCCTTGTTGATGTTTATTATTTATG 3'	31	BamHI
2.	MHR Reverse Primer	5' GCTAAGCTTTCAAACAGGACTATACATTCTGTCCT 3'	35	HindIII

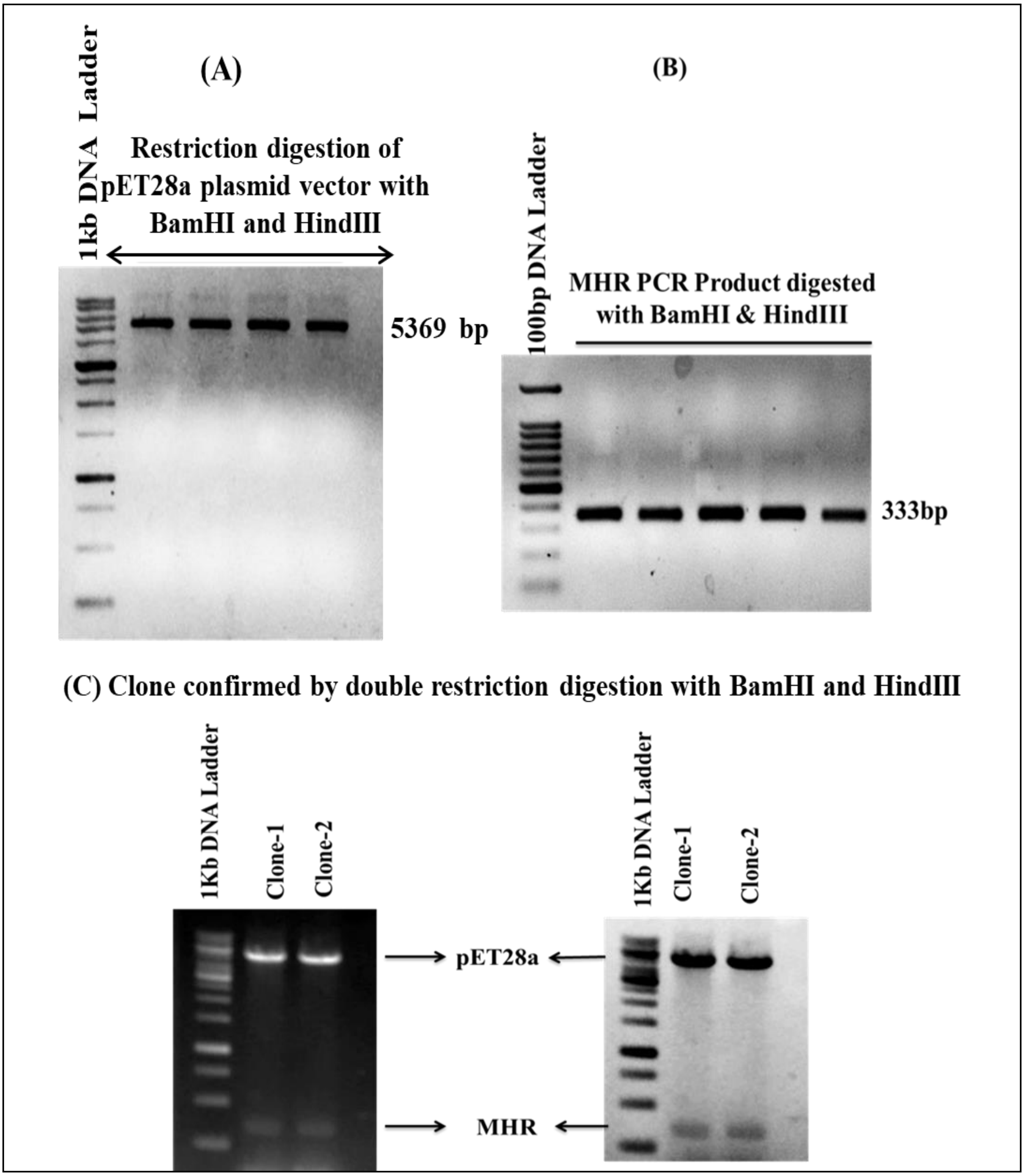

Figure S(2): Cloning of MHR domain in pET28a plasmid vector (A) Agarose gel electrophoresis of pET28a plasmid vector digested with BamHI & HindIII restriction enzymes. (B) Gel electrophoresis of the PCR product of MHR digested with BamHI & HindIII restriction enzymes. (C) Gel electrophoresis of clone confirmed with double restriction digestion with BamHI & HindIII restriction enzymes.
